# Drop-in biofuel production using fatty acid photodecarboxylase from *Chlorella variabilis* in the oleaginous yeast *Yarrowia lipolytica*

**DOI:** 10.1186/s13068-019-1542-4

**Published:** 2019-08-24

**Authors:** Stefan Bruder, Eva Johanna Moldenhauer, Robert Denis Lemke, Rodrigo Ledesma-Amaro, Johannes Kabisch

**Affiliations:** 1 Computer-Aided Synthetic Biology, Technische Universität Darmstadt, Schnittspahnstr. 12, 64287 Darmstadt, Germany; 2 Department of Bioengineering and Imperial College Centre for Synthetic Biology, Imperial College London, London, UK

**Keywords:** Drop-in biofuels, Clean fuels, Microbial biodiesel, Fatty acid photodecarboxylase, Hydrocarbons, Alkane, Alkene, Oleaginous yeast, *Yarrowia lipolytica*

## Abstract

**Background:**

Oleaginous yeasts are potent hosts for the renewable production of lipids and harbor great potential for derived products, such as biofuels. Several promising processes have been described that produce hydrocarbon drop-in biofuels based on fatty acid decarboxylation and fatty aldehyde decarbonylation. Unfortunately, besides fatty aldehyde toxicity and high reactivity, the most investigated enzyme, aldehyde-deformylating oxygenase, shows unfavorable catalytic properties which hindered high yields in previous metabolic engineering approaches.

**Results:**

To demonstrate an alternative alkane production pathway for oleaginous yeasts, we describe the production of diesel-like, odd-chain alkanes and alkenes, by heterologously expressing a recently discovered light-driven oxidase from *Chlorella variabilis* (CvFAP) in *Yarrowia lipolytica.* Initial experiments showed that only strains engineered to have an increased pool of free fatty acids were susceptible to sufficient decarboxylation. Providing these strains with glucose and light in a synthetic medium resulted in titers of 10.9 mg/L of hydrocarbons. Using custom 3D printed labware for lighting bioreactors, and an automated pulsed glycerol fed-batch strategy, intracellular titers of 58.7 mg/L were achieved. The production of odd-numbered alkanes and alkenes with a length of 17 and 15 carbons shown in previous studies could be confirmed.

**Conclusions:**

Oleaginous yeasts such as *Yarrowia lipolytica* can transform renewable resources such as glycerol into fatty acids and lipids. By heterologously expressing a fatty acid photodecarboxylase from the algae *Chlorella variabilis* hydrocarbons were produced in several scales from microwell plate to 400 mL bioreactors. The lighting turned out to be a crucial factor in terms of growth and hydrocarbon production, therefore, the evaluation of different conditions was an important step towards a tailor-made process. In general, the developed bioprocess shows a route to the renewable production of hydrocarbons for a variety of applications ranging from being substrates for further enzymatic or chemical modification or as a drop-in biofuel blend.

**Electronic supplementary material:**

The online version of this article (10.1186/s13068-019-1542-4) contains supplementary material, which is available to authorized users.

## Background

Modern human society is built upon readily available hydrocarbons, currently predominantly derived from fossil resources. The depletion of these as well as the adverse effects of their intense utilization has led to a variety of global challenges [1]. A concept to counteract these is to shift towards biobased processes by developing novel and drop-in alternatives produced on the basis of renewable resources. One such alternative are the so-called drop-in biofuels, which are substantially similar to current fuels and are not associated with some of the drawbacks of first-generation biofuels such as ethanol or fatty acid methyl esters [1]. These drawbacks include oxygen functional groups in the fuel molecules (e.g. fatty acid methyl ethers and ethanol) making them less infrastructure and engine compatible as well as the utilization of energy crops as substrates resulting in land-use changes [2].

In recent years, a variety of enzymes for the microbial production of hydrocarbons have been discovered and exploited. The most prominent among these are the pair formed by the acyl-ACP reductase (AAR) and the decarbonylating aldehyde-deformylating oxygenase (ADO) discovered in hydrocarbon producing cyanobacteria and expressed in *Escherichia coli* by Schirmer et al. [3]. Following this first proof of concept, the route from fatty acids to hydrocarbons was optimized and transferred to single cell oil (SCO) accumulating organisms [4].

Oleaginous yeasts are arbitrarily defined as being able to accumulate more than 20% of their cell dry weight (cdw) as lipids. Among these, the yeast *Yarrowia lipolytica* is well exploited with respect to genetic amenability and frequently used for industrial applications [5].

**Fig. 1:**
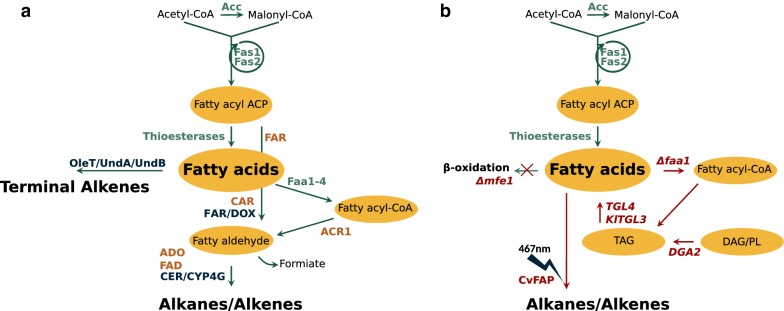
**a** Previously described pathways for hydrocarbon production with yeast (modified from [4]). *Y. lipolytica* enzymes are shown in green, intracellular metabolites in black. Orange coloured enzymes are investigated in [4], dark blue coloured enzymes are reviewed in [33]. Acc, acetyl-CoA carboxylase; Fas, Fatty acid synthase; AAR/FAR, fatty/acyl-ACP reductase; ADO, aldehyde-deformylating oxygenase; CAR, carboxylic acid reductase; DOX, α-dioxygenase; Faa, fatty acyl-CoA synthetase; ACR, fatty acyl-CoA reductase; FAD, fatty aldehyde decarbonylase; OleT, cytochrome P450 fatty acid decarboxylases (CYP152 family); UndA/B, aldehyde decarboxylases; CER/CYP4G, aldehyde decarbonylases. **b** Expression of CvFAP, with modifications of strain JMY5749 shown in red, characterized in this study. *DGA2*, acyl-CoA:diacylglycerol acyltransferase; *TGL4*/*KlTGL3* intracellular lipases; *MFE1*, peroxisomal multifunctional enzyme 1; CvFAP, fatty acid photodecarboxylase from *Chlorella variabilis*

The ability to produce large amounts of lipids makes it an attractive host for fatty acid-derived biofuels. Thus, the above-described pathways for hydrocarbon formation have been adapted to *Y. lipolytica* by Xu et al. [4]. Figure 1a summarizes different strategies for fatty acid-derived hydrocarbon formation with yeasts. A more recent publication identified a promiscuous activity of an algal photoenzyme [6]. This glucose–methanol–choline (GMC) oxidoreductase termed fatty acid photodecarboxylase (FAP) was found in both, *Chlorella variabilis* (CvFAP) and *Chlorella reinhardtii* (CrFAP).

The following study focuses on the production of hydrocarbons by expressing CvFAP in the oleaginous yeast *Y. lipolytica*. Initially, to extend the genetic accessibility of this unconventional yeast and simplify the cloning procedure, an in vivo cloning approach was established. In order to study the crucial role of the lighting and facilitate screening of different strain backgrounds, a medium throughput approach for determination of growth and hydrocarbon titer was set up. Finally, the production titer was maximized by optimization of a light-dependent bioreactor process.

## Results and discussion

Due to the low turnover number, the need of a coupled electron transfer system and the reactivity and toxicity of the intermediate fatty aldehydes, the expression of ADO in yeast was connected with high efforts of metabolic engineering but small hydrocarbon yields (Table 1). In contrast, the CvFAP enzyme directly utilizes free fatty acids (FFA) as its substrate as well as the readily available cofactor FAD. The catalysis is directly driven by the photons of blue light and hence tightly controllable. Unlike the AAR/ADO pathway, no additional genes for cofactor recycling are required [6]. Furthermore, a notably higher turnover number (8000 vs 0.0073 for substrates oleic acid and heptanal, respectively) was reported for the CvFAP [7, 8].

**Table 1:** Hydrocarbons produced by selected organisms, expressing different heterologous pathways

Pathway	Host	Titer	References
AAR/ACR/ADO	*Rhodococcus opacus*	5.2 g/L	[18]
AAR/ADO	*E. coli*	1.31 g/L	[34]
AAR/ADO	*E. coli*	~ 300 mg/L	[3]
FAP	*E. coli*	n.d.	[6]
CAR/ADO	*S. cerevisiae*	1.1 mg/L	[35]
OleT	*S. cerevisiae*	3.7 mg/L	[36]
UndB	*S. cerevisiae*	35.3 mg/L	[37]
DOX/ADO	*S. cerevisiae*	73.5 µg/L (304 µg/L by applying whole-cell biotransformation of pentadecanoic acid)	[38]
AAR(FAR)/ADO	*Y. lipolytica*	23.3 mg/L	[4]
FAP	*Y. lipolytica*	58.7 mg/L	This study

The enzyme abbreviations are namely *AAR* acyl-ACP reductase, *ACR* acyl-CoA reductase, *ADO* aldehyde-deformylating oxygenase, *FAP* fatty acid photodecarboxylase, *OleT* 450 fatty acid peroxygenase/decarboxylase, *UndB* desaturase-like enzyme, *DOX* α-dioxygenase

### Expression and characterization of CvFAP in *Yarrowia lipolytica* using YaliTAR

With regard to a rapid characterization, an in vivo DNA assembly strategy mediated by *Y. lipolytica* was carried out. In contrast to *Saccharomyces cerevisiae*, which mainly employs homologous recombination as a DNA repair mechanism, in *Y. lipolytica* non-homologous end-joining (NHEJ) is preferred [9]. As a consequence, several DNA assembly methods developed for baker’s yeast are not directly transferable. In previous studies, efficient homologous recombination for genomic integration with short length flanking fragments was successfully shown for *Y. lipolytica Δku70* mutant strains [10]. To transfer the frequently used baker’s yeast transformation-associated recombination (TAR) [11] for assembling the centromeric CvFAP expression plasmid within *Yarrowia*, co-transformation of the backbone and corresponding insert in a *Δku70*-strain background (H222 SW1) was successfully performed. The insert contains a *Y. lipolytica* codon-optimized sequence of the truncated CvFAP gene without signal peptide (Additional file 1: Seq. S1) flanked in front by a *TEF1* promoter and a *XPR2* terminator at the end.

Positive constructs (verified by sequencing) were grown on lipid body formation inducing YSM medium, containing 5% d-glucose as carbon source, underexposure with a readily available plant LED-light for 96 h. Under these light conditions, an intracellular titer of 112.1 ± 31.4 µg/L of hydrocarbons could be detected. In a dark experimental setup 1.5 ± 1 µg/L were detected. The empty vector control revealed no detectable production of hydrocarbons (Additional file 1: Table S1). The cloning method was coined as “YaliTAR”, derived from its *S. cerevisiae* analogon and enables direct characterization in *Y. lipolytica,* without the need for a shuttle host. The method can be generally applied for any other target gene and in particular deployed for rapid complementation of desired enzymatic activity.

### Alkane production with CvFAP in different *Y. lipolytica* strain backgrounds

To evaluate the influence of different strain backgrounds with respect to fatty acid availability, we transformed the replicative *C*vFAP expression vector into two different strains. We chose the laboratory strain H222 with knocked out beta-oxidation for increased lipid accumulation and deleted *ALK1* gene for inhibition of alkane degradation (S33001), as well as strain JMY5749 (Fig. 1b, Table 2), an overproducer of free fatty acids (FFA) [12] for enhanced substrate availability. A blue light LED-strip with a more distinct wavelength range (465–470 nm) was used. The duration of cultivation was 96 h to impede complete depletion of glucose to inhibit alkane degradation through C-catabolite repression [13]. The cell dry weights of both constructs were in a similar range at the end of the cultivations (3.6–4.4 mg/mL, Additional file 1: Table S2). A nearly 30 times higher hydrocarbon titer was achieved for JMY5749 compared to the S33001-strain background underexposure of blue light (1.551 ± 0.247 mg/L in contrast to 0.056 ± 0.004 mg/L, Additional file 1: Table S1). Despite the reduction of alkane monooxygenase activity [14], as well as incapacity of fatty acid degradation, the formation of hydrocarbons using S33001-strain background was lower than for JMY5749. In contrast, the latter features an increased lipase activity, and thus provision of substrate in higher intracellular concentrations, which underlines the requirement of the CvFAP for free fatty acids.

**Table 2:** *Yarrowia lipolytica* strains and constructs used in this study

Strain/constructs name	Genotype	Description	References
H222 SW1	H222 *MATA ura3::suc2 Δku70*	Improved homologous recombination capabilities	[9]
H222 Δku70/CvFAP	H222 MATA ura3::suc2 Δku70 including replicative plasmid p13001 (Additional file 1: Table S6)	Production of CvFAP in strain background mentioned above	This work
S33001	H222 *Δpox1*-*6 alk1Δ::mTFP Δura3*	Knocked out beta-oxidation for increased lipid accumulation and deleted *ALK1* gene for reduced alkane degradation	AG Kabisch, common strain collection
S33001/CvFAP	H222 *Δpox1*-*6 alk1Δ::mTFP Δura3* including replicative plasmid p13001 (Additional file 1: Table S6)	Production of CvFAP in strain background mentioned above	This work
S33001/empty vector control	H222 *Δpox1*-*6 alk1Δ::mTFP Δura3* including replicative plasmid p15018 (Additional file 1: Fig. S7)	Strain background mentioned above, transformed with empty vector control	This work
JMY5749	W29 *Δmfe1 Δfaa1 pTEF*-*DGA2 pTEF*-*TGL4 pTEF*-*KlTGL3*	FFA overproducing strain	Referred as JMY5479 in [12]
JMY5749/CvFAP	W29 *Δmfe1 Δfaa1 pTEF*-*DGA2 pTEF*-*TGL4 pTEF*-*KlTGL3* including replicative plasmid p13001 (Additional file 1: Table S6)	Production of CvFAP in FFA overproducing strain	This work
JMY5749/empty vector control	W29 *Δmfe1 Δfaa1 pTEF*-*DGA2 pTEF*-*TGL4 pTEF*-*KlTGL3* including replicative plasmid p15018 (Additional file 1: Fig. S7)	Strain background mentioned above, transformed with empty vector control	This work
S07003	JMY5749 *Δura3::54*-*55*	Partial deletion of *URA3* gene through a transformation with CRISPRyl-Hyg-URA3	This work
S07013	S07003 *Δura3* alk1::pTEF1-CvFAP(WT) *URA3*	Integration of CvFAP cassette obtained from p13012	This work
S07004	S07003 *Δura3* alk1::pTEF1-CvFAP(S121F) *URA3*	Integration of CvFAP cassette obtained from p13012	This work

**Fig. 2:**
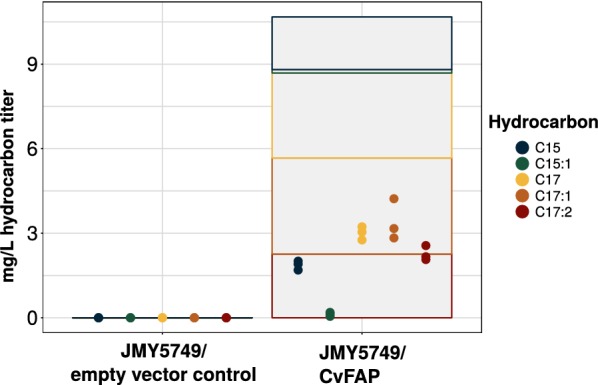
Alkane production with CvFAP expressed in *Yarrowia* JMY5749 in contrast to empty vector control (Strain background described in Table 2, medium composition in “Materials and methods” section). The intracellular titer of each hydrocarbon is indicated by dots (in triplicate), the sum of hydrocarbons and the hydrocarbon composition is represented by a bar plot

By optimizing the extraction method through the usage of a ball mill, lowering sample volume and dilution of the sample (see “Material and methods” section), a titer of 10.87 ± 1.11 mg/L total hydrocarbons could be detected, using JMY5749/CvFAP (shown in Fig. 2). Most of the hydrocarbons produced were heptadecane, 8-heptadecene and 6,9-heptadecadiene in similar levels followed by pentadecane and 7-pentadecene. Furthermore, the measurement of total fatty acids revealed a significant lower intracellular titer of 35 mg/g to 21 mg/g compared to the empty vector control (Additional file 1: Fig. S1). The hydrocarbon spectrum, listed above, is in accordance with previous findings using *E. coli* [6] and in vitro experiments [8].

The empty vector control did not show any hydrocarbon formation nor could hydrocarbons (alkanes C8–C20) be detected in the supernatants of any samples (data not shown).

### Examination of process parameters and strain development using a custom-made device for cultivations in 24-well scale

**Fig. 3:**
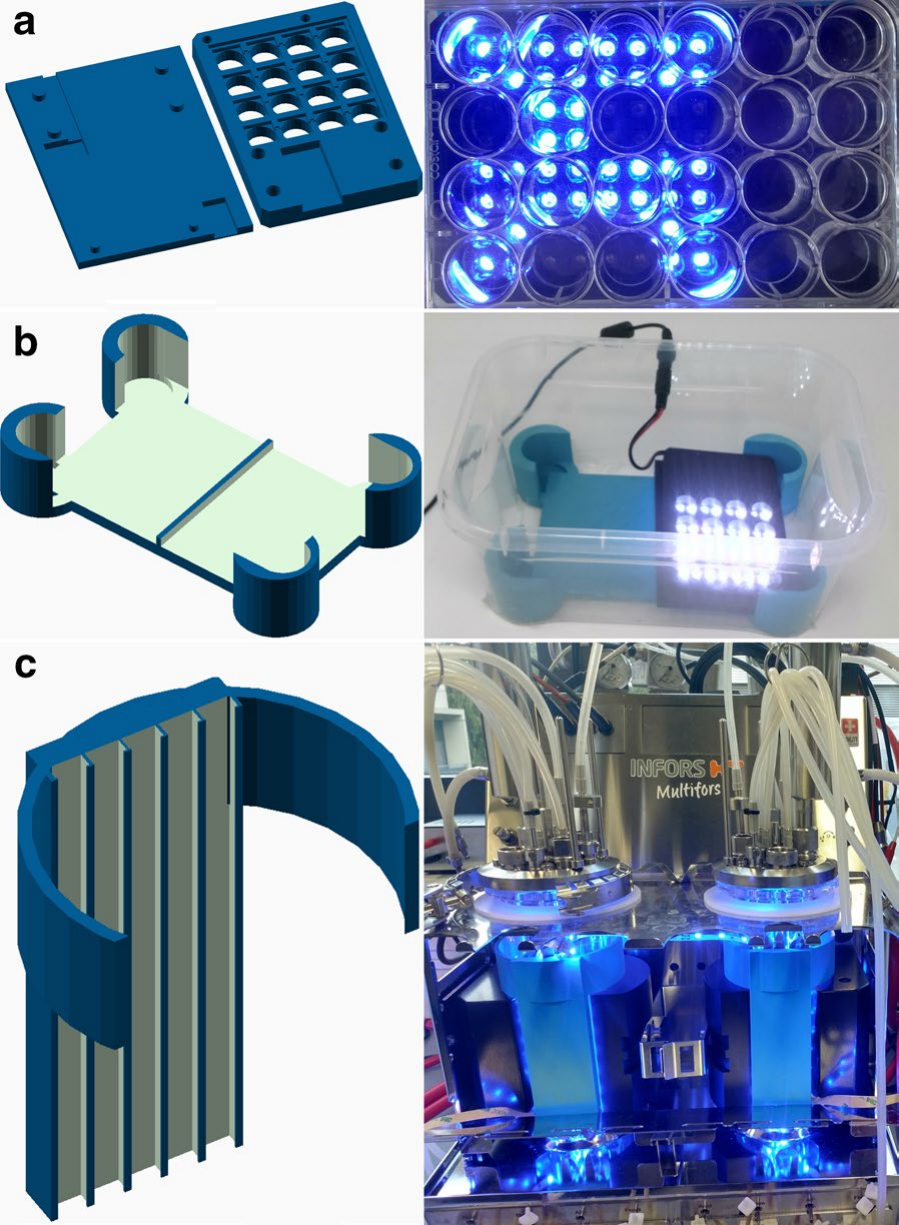
3D printed custom labware for evaluation of light regimes. **a** Rendering and picture of a LED-matrix plate for testing light regimes in microwell plates. **b** Low evaporation setup with custom microwell plate-holder and LED-matrix in a low-cost plastic box. **c** Rendering and picture of LED-strip-holder attached to bioreactors

To examine the light intensity, duration of exposure or effect of light pulsing, a medium-throughput approach for cultivations in 24-well plates was established. Besides the tracking of optical density, the cultivation volume of 750 µL was sufficient to allow endpoint measurement of intracellular hydrocarbons. Originating from a custom-made LED-device [15], adapters for LED-matrix and 24-well plates, as well as a universal plate-holder for incubators were fabricated (Fig. 3a, b). The rapid prototyping of custom labware proofed to be a very valuable tool in this work. Using free, open-source software such as openSCAD, readily available designs from previous publications and a 3D printer, we were able to parallelize the workflow using the 24-well LED plates in a shaker and avoid evaporation (data not shown) without resorting to expensive, commercial solutions.

**Fig. 4:**
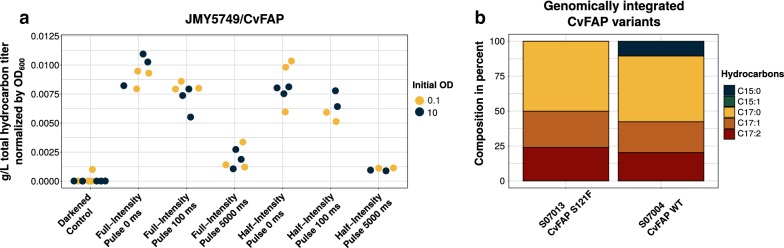
**a** Endpoint measurements of total hydrocarbons formed in microwells with different light regimes. Inoculation with high (set OD_600_ of 10; blue dots) and low (OD_600_ was set to 0.1; yellow dots) initial cell densities. Full light intensity was determined as 28.7–32.3 µmol quanta m^−2^ s^−1^ per well. Light regimes were tested in triplicates, except for half intensity, pulse 100 and 5000 ms, which were cultivated in duplicates. **b** Alkane/alkene compositions of endpoint measurements for cultivations (initial OD_600_ 0.1) of two clones, harboring the genomic integration of different *Cv*FAP variants. Strains were cultivated in triplicates, expect for *n* = 2 S07013

Cultivation of the strain JMY5749/CvFAP (JMY5749 transformed with p13001, Table 2) at high cell densities on glucose-containing YSM medium showed the highest intracellular hydrocarbon titer using maximum LED intensity of 29–32 µmol quanta m^−2^ s^−1^ per well and continuous lighting. Short light pulses with breaks of 100 ms or 5000 ms, as well as an intensity reduced by half, led to significantly reduced hydrocarbon formation (Fig. 4a blue dots, Additional file 1: Table S3). The growth, determined by optical density, was not affected by any of the lighting conditions (Additional file 1: Fig. S2A). To further investigate a putative low impact of continuous lighting or even pulsing, the measurements were repeated by adjusting the initial high OD_600_ to 0.1 (Fig. 4a, yellow dots). Again, a reduction of the growth rates at given intensities could not be detected (Additional file 1: Fig. S2B). In contrast to the first approach, the lighting with half intensity led to a similar hydrocarbon titer, which indicates the importance of fine-tuning light exposure and cell growth (Additional file 1: Table S4).

To examine further genetic contexts, strains harboring a genomic integration of CvFAP were characterized with the help of the 24-well LED-device. While the sequence of the CvFAP coding region was correct in most of the investigated clones, a spontaneous mutation (S121F in WT, S61F for truncated *Cv*FAP without signal peptide) occurred for strain S07004. To characterize the putative implications on hydrocarbon composition, strain S070013 harbouring WT CvFAP as well as S07004 harbouring the variant (CvFAP S121F) were both cultivated. By applying continuous lighting at highest possible intensity, both clones showed similar hydrocarbon titer after 96 h of cultivation in YSM medium (Additional file 1: Fig. S2C) but revealed different hydrocarbon compositions (Fig. 4b). For a more precise characterization, the strains were examined in bioreactor cultivations.

### Characterization of different strains and light intensities, using bench-scale bioreactors

To perform light-dependent bioreactor cultivations, custom-made LED-strip holders for the reactor vessels were fabricated using 3D printing (Fig. 3c). The construction of the holding clips attaching the LED-strips to the bioreactors ensure reproducible lighting conditions (487–560 quanta photons m^−2^ s^−1^ for full light intensity). Batch medium contained 30 g/L glycerol as carbon source and 5 g/L ammonium sulfate for the generation of biomass. A pulse of 30 g/L C-source containing feed solution was added when C-source was depleted [detected by the rise of dissolved oxygen (DO)]. By omitting a nitrogen source in the feed medium, an increased C/N ratio and thus an increased formation of free fatty acids should be achieved [12]. The compositions of batch and feed medium, as well as a detailed description of the bioreactor and cultivation conditions are listed in “Materials and methods” section. The sequence for DO-dependent automated feeding is listed in Additional file 1: Seq. S2).

To analyse the impact of the CvFAP variant, clones S07004 (CvFAP S121F) and S07013 (CvFAP WT) were cultivated in triplicates using the LED-equipped bioreactors. In contrast to previous experiments, glycerol was selected as carbon source, in particular due to its availability as a sidestream in biodiesel production. While bioreactor parameters, as well as hydrocarbon composition were similar, strain S07004 performed slightly better than strain S07013 in terms of intracellular total hydrocarbon titer and cell dry weight (Additional file 1: Fig. S3). In contrast to the hydrocarbon composition obtained in the 24-well plates, pentadecane could be detected after 15 h of cultivations in all bioprocesses of both, wild type and the S121F variant. These differences between the bioreactor and 24-well plates could be caused by threefold increased light intensity (max. 60–90 µm quanta m^−2^ s^−1^) in the bioreactor. The findings underline that while the microwell cultivations can be used as a first screening a controlled process environment is required for in-depth analysis.

Based on the better performance of S07004 further studies were continued with this strain harboring the amino acid exchange. An in silico structural analysis of the CvFAP S121F variant with the published CvFAP WT structure revealed a minimum distance of 12 Å, between the phenylalanine ring and flavin adenine dinucleotide, but was shielded by secondary structures (Additional file 1: Fig. S4). According to literature, different positioning of the functional carboxyl group to the cofactor could have a strong influence on substrates conversion rates or yields [8]. Thus, an indirect influence due to altered coordination of intermediate aa residues should be examined in future studies.

#### Improvement of hydrocarbon production depends on the orchestration of light intensity and growth

In the 24-well experiments, no reduction in growth could be detected by using a maximum intensity of 32 µmol quanta m^−2^ s^−1^ per well. The LED-strips attached to the bioreactors allowed for a roughly twenty times higher light intensity (approx. 560 µmol quanta m^−2^ s^−1^).

**Fig. 5:**
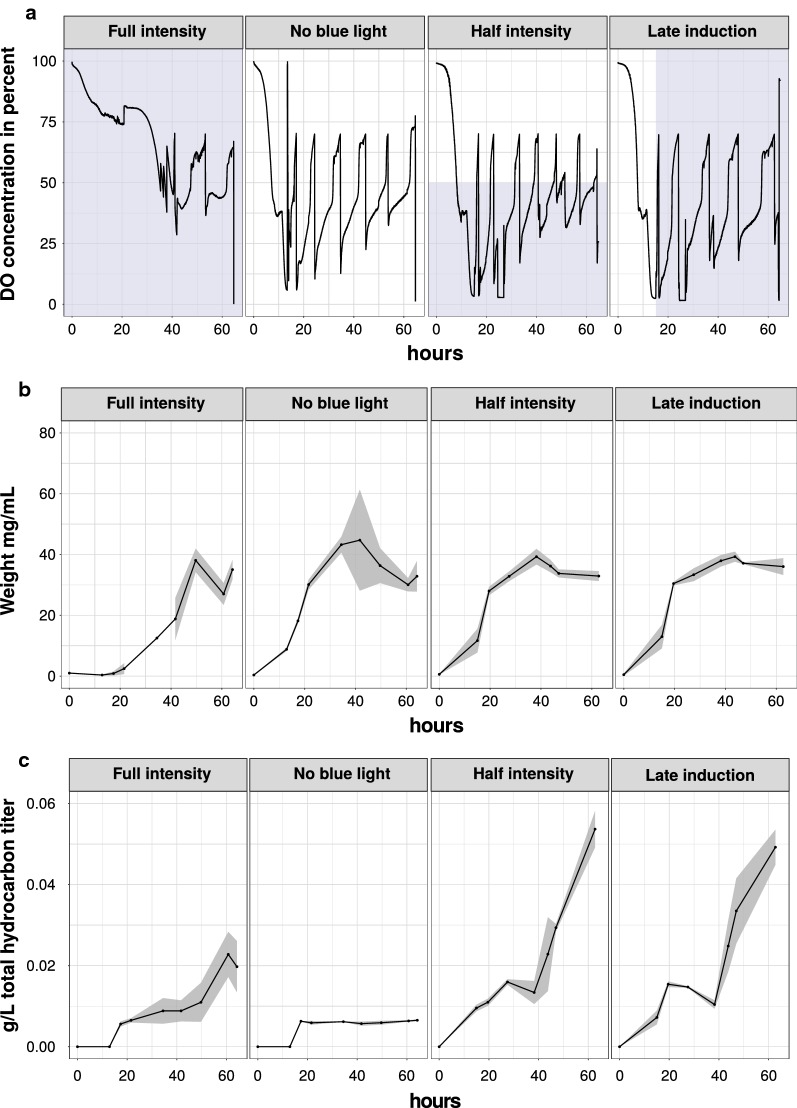
Bioprocesses with four different light regimes were characterized in triplicates by the cultivation of strain S07004. Light intensity (height) and exposure time (width) are indicated as blue shaded areas. For full intensity, the light intensity was set to 545 µm quanta m^−2^ s^−1^, while half intensity reached 250 µm quanta m^−2^ s^−1^. For the no blue light control, fermenter vessels were shielded from neighboring blue light but were still affected by ambient light. For the late induction experiment, full light intensity was switched on 16 h after inoculation. **a** DO concentration in the percentage of representative cultivations. **b** Cell dry weight measurements in mg/L of the bioreactor cultivations in triplicate were joined and the deviation of the mean is indicated by ribbons. **c** Total intracellular hydrocarbon titer determined in triplicate

To obtain the highest amount of total hydrocarbons, four different light settings were evaluated. In addition to full intensity and no blue light control (ambient light), half intensity (approx. 200 μmol quanta m^−2^ s^−1^) and induction with full intensity 16 h after inoculation were also tested (Fig. 5; Additional file 1: Fig. S5). The values of the DO-probe were used as an online measurement of the metabolic activity of the cells (Fig. 5a). Full intensity led to a decreased amount of feeding cycles and a longer interval between each feeding pulse in comparison to the no-light, half-intensity and late-light induction processes (Fig. 5a). In contrast to the other conditions and despite similar levels of biomass formation, intracellular octadecanoic acid concentration was elevated (Additional file 1: Fig. S5A1,2) during full light conditions. Furthermore, the reduced feeding was accompanied by the lower formation of extracellular metabolites such as citrate and polyols (Additional file 1: Fig. S5B1,2). Interestingly, the highest total hydrocarbon formation with regard to the cell dry weight, 1.26 ± 1.19% (0.80%, 2.5%, 0.36%), could be obtained after 17 h of cultivation. On average, the remaining light-dependent bioprocesses revealed a maximum total hydrocarbon formation of 0.14% cdw (half of intensity: 0.169%, 0.151%, 0.168%; late induction: 0.081%, 0.158%, 0.111%), primarily resulting from the more rapid increase of the biomass (Fig. 5b). The severe growth impairment with full light conditions from the onset of the bioprocess suggest that the CvFAP sequesters most fatty acids formed. After almost 20 h of lag-phase the cells seem to recover and resume growth, but stop producing hydrocarbons. In literature, high light intensity, especially irradiation in the range of 450 nm, is linked to a phenomenon called photoinactivation, which results in *S. cerevisiae* cells that are in a viable, but nonculturable state. As photosensitizers, flavins and porphyrins are discussed [16]. Furthermore, especially for blue light, a significant effect on yeasts respiratory oscillation is described [17]. To what degree these findings are transferable to *Y. lipolytica* needs to be examined in future studies.

Considering the absolute total hydrocarbon formation, best results were achieved using half light intensity with a maximum of 58.69 mg/L, closely followed by late induction experiments (52.23 mg/L). To our knowledge and in comparison to other studies harnessing *Y. lipolytica* or other yeasts as host organisms, these are the highest hydrocarbon titer described so far. Still, by implementing the AAR/ADO pathway into *E. coli* or an oleaginous bacterium (see Table 1 for comparison with recent studies) higher titers could be reached [18].

A decrease in hydrocarbon production in the form of stagnating or declining values was observed within all light processes. This indicates a putative degradation of formed alkanes or alkenes. In *Y. lipolytica*, the presence of *n*-alkanes leads to transcriptional activation of alkane-degrading enzymes. The main monooxygenase (*ALK1*) responsible for hydrocarbon degradation [19] was deleted, but the remaining *ALK2*-*12* enzymes are still sufficient for the degradation of long-chain hydrocarbons [20]. Nevertheless, the corresponding genes are subject to transcriptional repression due to glycerol feeding [19]. For half intensity and late induction settings, a major decrease could be detected after 40 h of cultivation. This coincides with a temporary glycerol exhaustion due to the broad settings of saturated oxygen limits of the feeding sequence (< 40%, > 70%; shown in Additional file 1: Seq. S2—compare with extracellular glycerol titers, shown in Additional file 1: Fig. S5B1, 2). Consequently, a tighter process control, taking into account the disadvantage of adaptation for specific strain backgrounds and light regimes, as well as further deletions of genes encoding alkane-degrading enzymes are likely candidates for further improvement of the hydrocarbon titer.

**Fig. 6:**
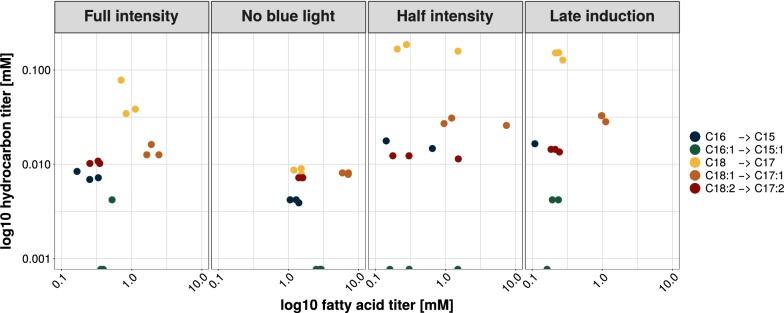
Amounts of fatty acids and hydrocarbons formed until the end of the cultivation. Axis are scaled logarithmic (base 10). Time-resolved values are shown in Additional file 1: Fig. S6

Generally, in all light-induced bioprocesses, with the exception of the no blue light control, a predominant formation of heptadecane (C17:0) occurred (Additional file 1: Fig. S5C1,2). In no blue light control (ambient light), similar amounts of C17:0 and unsaturated 8-heptadecene (C17:1) as well as 6,9-heptadecadiene (C17:2) were detected. Considering the fatty acid composition, this stands in contrast to the predominance of unsaturated fatty acids such as for example oleic acid (C18:1) over octadecanoic acid (C18:0). Thus, a preference of the CvFAP in *Y. lipolytica* for saturated fatty acids can be assumed. This holds true for all detected fatty acids as shown in Fig. 6 and time-resolved in Additional file 1: Fig. S6. While the lowest intracellular fatty acid titers could be assigned to hexadecanoic acid (C16:0), converted pentadecane (C15:0) partially showed the third-highest titers of detected hydrocarbons. In contrast, the highest intracellular fatty acid titer could be assigned to oleic acid (C18:1) but the converted 8-heptadecene (C17:1) were only the second-highest hydrocarbon titer detected. The findings were similar for linoleic acid (C18:2) and derived 6,9-heptadecadiene (C17:2). Values of palmitoleic acid in comparison to 7-pentadecene (C16:1, C15:1) imply the lowest conversion. In the no blue light control these effects were not fully confirmed (Additional file 1: Fig. S6). The preference for saturated fatty acids are in accordance with the findings for purified CvFAP enzyme, which exhibits higher conversion rates for saturated fatty acids [8].

## Conclusions

Expression of CvFAP in oleaginous, fatty acid-secreting *Yarrowia lipolytica* under blue light exposure leads to the production of odd-numbered alkanes and alkenes with a predominant length of 17 and 15 carbons. Especially the absence of reliable and readily available, inducible promoters for *Y. lipolytica* makes this light-driven reaction desirable with respect to process control. By omitting the inducing wavelength, the enzyme could be produced from a constitutive promoter and catalysis was switched on only after sufficient amounts of fatty acids have accumulated. 3D printing and readily available LED technology are especially interesting technologies to combine with light-driven bioprocesses, enabling researchers to rapidly develop custom labware.

Future strain engineering should include aspects such as increasing gene copy numbers, reduce secreted metabolites and modify fatty acid profiles. Process designs should take carbon dioxide (CO_2_) released during decarboxylation into consideration and could include recently described CO_2_ fixation approaches [21, 22].

## Materials and methods

### Transformation-associated recombination assisted by *Y. lipolytica* (YaliTAR) for rapid construction of simple replicative vectors

For time-saving in vivo assembly, Yarrowia strain H222 *Δku70* was co-transformed [23] with linearized replicative vector p15018 backbone (Additional file 1: Fig. S7A), digested by MluI and NotI, as well as codon-optimized CvFAP fragment (Additional file 1: Seq. S1), including 43 bp homologous sequences to *TEF1* promoter (additional 6 bp MluI restriction site for further promoter exchanges) and *XPR2* terminator of p15018. The coding sequence of the CvFAP was synthesized by Baseclear B.V., without the predicted targeting sequence as shown in [6]. Oligonucleotides for amplification of overlapping fragments are listed in Additional file 1: Table S5. Positive clones of the resulting plasmid p13001 were selected on YPD2% agar plates including 400 µg/mL hygromycin after 1–2 days of incubation at 30 °C. Vectors, recovered from 4 out of 14 colonies, were verified by sequencing, whereby 50% showed the correct sequence. YaliTAR method was also applied for the exchange of a marker gene of the Cas9 expression vector pCRISPRyl [24], which was provided by Ian Wheeldon (Addgene plasmid # 70007). Exchange from leucine to hygromycin marker resulted in vector p55001, verified by sequencing. Further integration of sgRNA was performed using SLiCE in vitro method, described below (oligos listed in Additional file 1: Table S5, vectors in Additional file 1: Table S6). *Yarrowia* strains H222 *Δku70*, S33001 and JMY5749 (Table 2) were transformed with p13001 vector.

### *Y. lipolytica* strain construction

The backbone, originating from integrative vector p33001 (Additional file 1: Fig. S7B), and CvFAP cassette from p13001 were amplified with overlapping homologous overhangs (Additional file 1: Tables S5, S6). For the assembly of both parts, SLiCE method was used, described by [25] including minor deviations described in [26]. This resulted in vector p13012. CRISPRyl-Hyg-URA3 (p94001) was constructed likewise, sgRNA was designed by CHOPCHOP v2 online tool [27, 28]. Deletion of *URA3* by transformation with p94001 and counter selection with 5-fluoroorotic acid [29] of strain JMY5749 yielded the required auxotrophy for the successful integration of CvFAP into the *ALK1* locus of the JMY5749 genome. To verify the integration transformed cells were selected by growth on YNB ura^−^ plates. Positive clones were picked and checked by sequencing.

### Cultivation conditions and sampling for shake flask experiments

5 mL YPD2% (referred elsewhere) for inoculation and 25 mL YSM medium (*Yarrowia* low mineral Salt Medium) for cultivation were used. YSM was designed for the induction of lipid droplet (LD) formation in fed-batch cultivations based on [30, 31]. The medium was composed as a cost-effective alternative to common LD inducible media and consists of the compounds listed below. Base compounds: 1.6 g/L Na_2_HPO_4_·2H_2_O, 0.092 g/L KH_2_PO4, 0.5 g/L (NH_4_)_2_SO_4_; further supplements: 0.7 g/L MgSO_4_·7H_2_O, 0.1 g/L CaCl_2_·6H_2_O, 0.5 g/L yeast extract, 50 g/L d-glucose; trace elements: 0.5 mg/L H_3_BO_3_, 0.4 mg/L MnSO_4_·H_2_O, 0.4 mg/L MnSO_4_·H_2_O, 6 mg/L FeCl_3_·6H_2_O.

Cultivations in shake flasks were performed at RT (H222 *Δku70*/CvFAP and empty vector control) or 28 °C and 180 rpm. The shakers were darkened as indicated. Light for the photoenzyme was provided by a commercial blue light LED-strip with an advertised wavelength of 465–470 nm (Additional file 1: Fig. S8) or a common LED plant breeding light from Florally Inc. (Shenzhen, Guangdong, 518000, CN). Samples were taken after 96 h for the determination of cell dry weight, intracellular and extracellular hydrocarbons and metabolites in the supernatant. For analytics, the entire volume of supernatant and cells was utilized (for improved extraction method, 1 mL of sample volume was chosen).

### Cultivation conditions and sampling in a custom-made 24-well device

For testing the influence of different intensities and pulsing of the blue light required for photodecarboxylation a LED-matrix plate and holder were fabricated using 3D printing. The setup is depicted in Fig. 3a, b, design and printing is described in “Materials and methods”—“Design and printing of custom labware”. For incubation in a shaker, a common plastic box with dimensions width/depth/height of 12.1/25.5/13.6 cm (RegaLux clear Box XXS, BAUHAUS, Switzerland), as well as 24-well sensor plates with glass bottom and darkened walls (Sensoplate glass bottom, Black, Greiner BIO-ONE, Austria) were used. Cells were grown on 750 µL YSM medium at 28 °C and 180 rpm. For endpoint measurements, residual volume of cultivation broth was measured and used for hydrocarbon analytics, described below.

### Bioreactor cultivations and sampling

Batch medium for fermentation contained 30 g/L carbon source (glucose or glycerol), 0.5 g/L yeast extract, 1.1 g/L MgSO_4_*7H_2_O, 0.2 g/L CaCl_2_*6H_2_O, 0.5 g/L MgCl_2_*6H_2_O, 0.075 g/L myo-Inositol, 1.36 g/L KH_2_PO_4_, 1.74 g/L K_2_HPO_4_, 0.2 mg/L CuSO_4_*5H_2_O, 1 mg/L FeSO_4_*7H_2_O, 0.2 mg/L MnCl_2_*4H_2_O, 0.2 mg/L Na_2_MoO_4_*2H_2_O, 0.2 mg/L ZnSO_4_*7H_2_O, 5 mg/L biotin, 100 mg/L D-pantothenic acid hemicalcium salt, 20 mg/L nicotinic acid, 60.8 mg/L pyridoxine hydrochloride, 20 mg/L thiamine hydrochloride and 5 g/L NH_4_Cl. Feed medium for fermentation contained 400 g/L carbon source (glucose or glycerol), 3.3 g/L MgSO_4_*7H_2_O, 0.6 g/L CaCl_2_*6H_2_O, 1.5 g/L MgCl_2_*6H_2_O, 0.45 g/L meso-Inositol, 2.72 g/L KH_2_PO_4_, 3.48 g/L K_2_HPO_4_, 0.6 mg/L CuSO_4_*5H_2_O, 3 mg/L FeSO_4_*7H_2_O, 0.6 mg/L MnCl_2_*4H_2_O, 0.6 mg/L Na_2_MoO_4_*2H_2_O, 0.6 mg/L ZnSO_4_*7H_2_O, 15 mg/L biotin, 300 mg/L d-pantothenic acid hemicalcium salt, 60 mg/L nicotinic acid, 182.4 mg/L pyridoxine hydrochloride, 60 mg/L thiamine hydrochloride and 0.01 g/L FeCl_3_*6H_2_O. Bioreactors (Infors multifors 2) were inoculated to an optical density at 600 nm of 0.1 in 300 mL of batch medium from overnight shake flask. Initial process parameters were pH 6.0, temperature of 30 °C, aeration at 1 lpm air, and agitation at 400 rpm. Due to the production of citric acid by the host, pH drops to a value of below 4 in the early phase of the cultivation. Afterwards, the pH was adjusted automatically to 4.0 or higher by 2 N sodium hydroxide and the agitation was adjusted to up to 1000 rpm depending on the dissolved oxygen (DO) concentration. A pulse of feed medium (volume corresponding to 30 g C-source per liter initial batch volume) was automatically supplied whenever the C-source was consumed (detected by the increase of DO). Samples for GC-FID analysis of hydrocarbons and fatty acid composition as well as cdw were taken periodically. Samples for the determination of the cdw were centrifuged at 16,000×*g*, 5 min and dried at 60 °C at least 24 h until complete dryness. The cdw was determined gravimetrically. The intensity of the attached LED-stripes was controlled by a standard laboratory power supply. The ratio of single LED-light intensity and the current was determined (Additional file 1: Table S7) to facilitate reproducible blue light intensities.

### Lipid extraction, transesterification for GC analysis

For the analysis of lipid content, samples of 1 mL culture volume (or different when indicated) were taken during cultivations and centrifuged at 16,000×*g*, 5 min. Cell pellets were washed with 1 mL of deionized water followed by a second centrifugation step. Cells were resuspended in 200 µL deionized water and 200 µL glass beads (1:1 mixture of diameters of 0.25–0.5 mm and 0.1 mm) were added to the suspension, as well as 300 µL of n-hexane:2-propanol 3:1 containing internal standard (5 mM tridecanoic acid) for extraction of triacylglycerols (TAG). Cell lysis was performed in a ball mill (Mixer Mill MM 400) at 30 Hz for 20 min. The lysate was centrifuged at 16,000×*g* for 1 min and the upper organic phase was transferred to a glass vial. To remove residual water, 50 µL of 2,2-dimethoxypropane were added. Transesterification was performed by the addition of 500 µL 2% (v/v) methanolic H_2_SO_4_ and incubation at 60 °C and 1400 rpm in an Eppendorf Thermomixer comfort for 2 h. After extraction in 300 µL of n-hexane and optional desiccation over sodium sulfate, the fatty acid methyl ester (FAME) solution was stored at − 20 °C until gas chromatography (GC) analysis. For peak assignment, FAME mix from Sigma Aldrich (CRM18918) was used. For quantification, a standard curve of the single FAMEs from Sigma Aldrich Fluka in a concentration range of 0.025–8 mM were recorded. The samples were analyzed with a Shimadzu Nexis GC 2030, on a Shimadzu SH-Rxi-5MS column (30 m, 0.25 mm, 0.25 µm) and detected by FID. The temperature of inlet and FID were set to 250 °C and 310 °C, respectively. The linear velocity of hydrogen was set to 50 cm/s. Split to 10. Column oven temperature program: Temp. 90 °C, hold 5 min; Rate 15, final temp. 190 °C; Rate 2.0, final temp. 200 °C, hold 1 min; Rate 0.5, final temp. 202.5 °C, hold 1 min; Rate 20, final temp. 300 °C, hold 5 min. Data were processed using LabSolutions 5.92 and R version 3.4.4 (2018-03-15) as well as RStudio 1.2.1335.

### Hydrocarbon analytics of cell extract and supernatant

Analysis was similar to the lipid protocol, including first washing step. Cell lysis was performed, using a Vortexer (10 min, 3000 rpm) from Heathrow Scientific (indicated as non-optimized extraction method in Additional file 1: Tables S1, S2). For optimized extraction/lysis, a ball mill (Mixer Mill MM 400) at 30 Hz for 20 min was used. The lysate was centrifuged at 16,000×*g* for 1 min and the upper organic phase was transferred to a glass vial. For cell extraction, 300 µL *n*-Hexane containing 5 mM *n*-dodecane as internal standard, for extraction of whole supernatant (shake flasks experiments) 1.8 mL *n*-hexane containing 5 mM n-dodecane was used. Detection of hydrocarbons was performed using gas chromatography. GC settings are previously described. The split was set to 50 for samples of total cell extractions from shake flasks, to 5 for 1 mL samples from shake flasks, 24-well and bioreactor measurements and to 10 for samples of the extracted supernatant. The temperature profile was set to an initial temperature of 50 °C, which was held for 2.5 min, followed by a ramp to 250 °C at a rate of 10 °C per min, followed by a ramp to 300 °C at a rate of 10 °C per min and a final step at 300 °C for 10 min. The analytical GC grade standards undecane, tridecane, pentadecane, heptadecane and the C8–C20 alkane standard solution were purchased at Sigma Aldrich. Quantification of 7-pentadecene, 8-heptadecene and 6,9-heptadecadiene were performed according to [32]. Corresponding peaks were clearly distinguishable from background noise (Additional file 1: Fig. S9AB). MS spectra, using Shimadzu GCMS QP2010 and column BPX5 (Column has equal properties to SH-Rxi-5MS, but the retention time was slightly shifted, the program is described above) from selected samples were compared to NIST database (GCMSsolution version 4.42, NIST) and confirmed the presence of saturated pentadecane (97% similarity), 8-heptadecene (91% similarity) and heptadecane (93% similarity). The retention time difference between monounsaturated 1-pentadecene standard (15.969 min, 98% similarity) and saturated pentadecane (16.049 min) as well as between 8-heptadecene (18.152 min) and heptadecane (18.433 min) further confirmed the above assumed assignments. Data conversion and plotting were performed with R as described above.

### HPLC analysis of extracellular metabolites and media components

Samples were filtered using 10 K modified PES Centrifugal filters (VWR). Metabolites of remaining cell-free supernatant were analyzed by HPLC. Concentrations of d-glucose, citrate and polyols were determined by Perkin Elmer Series 200, using a RezexTM ROA-Organic Acid H+ (8%) column (Phenomenex, California, USA). References were purchased from Sigma Aldrich. The column was eluted with 5 mM sulfuric acid as mobile phase and a flow rate of 0.4 mL/min at 65 °C. Refractive index values were detected by RI-101 (Shodex). For data evaluation, TotalChrom Workstation/Navigator software (Perkin Elmer, version 6.3.2) was used. Data conversion and plotting were performed with R as described above.

### Design and printing of custom labware

CAD for the custom labware was performed using OpenSCAD version 2015.03-1. The basic design of the 24-well plates was kindly provided by the Möglich lab and is based on their previously published 96-well plates [15]. This design was modified in tinkercad, to fit into our microwell holders as well as to accommodate an easier electronic setup by using a FadeCandy (Adafruit Part Number: 1689) driver for the LED-matrix (Adafruit NeoPixel, Adafruit industries, New York, USA). Slicing for 3D printing was performed using Simplify 3D version 4.0.1. The labware was printed on a Makergear M2 using PLA as filament.

## Additional file

**Additional file 1: Seq. S1.** Sequence of truncated CvFAP, optimized for *Y. lipolytica* codon usage. **Table S1.**Characterization of H222 *Δku70*, JMY5749, S33001. **Table S2.** Addendum for characterization of JMY5749 and S33001. **Fig. S1.** Intracellular fatty acid concentrations of *Yarrowia* JMY5749/CvFAP constructs and empty vector control. **Table S3.** Results of Anova method for coefficients shown in Fig. 4 A. **Fig. S2.** Impact of different light regimes on cultivations of JMY5749/CvFAP. **Table S4.** Results of Anova method for coefficients shown in Fig. 4B. **Seq. S2.** Sequence for DO-dependent automated feeding. **Fig. S3.** Comparison of CvFAP variant (S07004, S121F) and wild type (S07013), cultivated in triplicates. **Fig. S4.** Rendering of CvFAP WT and S121F variant. **Fig. S5.** Additional parameters of bioprocesses with four different light regimes (Fig. 5). **Fig. S6.** Preferred conversion of available substrates to hydrocarbons. **Fig. S7.** Vector maps of plasmids p15018 and p33001. **Table S5.** List of oligonucleotides. **Table S6.** List of constructed vectors. **Fig. S8.** Light emission of LED-device. **Table S7.** Correlation of light intensity and power supply values. **Fig. S9.** Hydrocarbon peaks vs background.

## Data Availability

All 3D designs are available in our gitlab repository https://gitlab.com/kabischlab.de/led-labware-cvfap/

## Abbreviations

Aa: amino acid
AAR (FAR): (fatty) acyl-ACP reductase
ADO: aldehyde-deformylating oxygenase
CAR: carboxylic acid reductase
cdw: cell dry weight
*Cv/r*: *Chlorella variabilis/reinhardtii*
DO: dissolved oxygen
DOX: α-dioxygenase
FFA: free fatty acids
GMC: glucose–methanol–choline
SCO: single cell oil
FAP: fatty acid photodecarboxylase
RT: room temperature
YaliTAR: transformation-associated recombination assisted by *Y. lipolytica*
WT: wild type

## References

[1] Junne S, Kabisch J (2017). Fueling the future with biomass: processes and pathways for a sustainable supply of hydrocarbon fuels and biogas. Eng Life Sci.

[2] Yan J (2015). First-generation biofuels. Handbook of clean energy systems.

[3] Schirmer A, Rude MA, Li X, Popova E, del Cardayre SB (2010). Microbial biosynthesis of alkanes. Science.

[4] Xu P, Qiao K, Ahn WS, Stephanopoulos G (2016). Engineering *Yarrowia lipolytica* as a platform for synthesis of drop-in transportation fuels and oleochemicals. Proc Natl Acad Sci USA.

[5] Larroude M, Rossignol T, Nicaud J-M, Ledesma-Amaro R (2018). Synthetic biology tools for engineering *Yarrowia lipolytica*. Biotechnol Adv.

[6] Sorigué D, Légeret B, Cuiné S, Blangy S, Moulin S, Billon E (2017). An algal photoenzyme converts fatty acids to hydrocarbons. Science.

[7] Zhang J, Lu X, Li J-J (2013). Conversion of fatty aldehydes into alk (a/e)nes by in vitro reconstituted cyanobacterial aldehyde-deformylating oxygenase with the cognate electron transfer system. Biotechnol Biofuels.

[8] Huijbers MME, Zhang W, Tonin F, Hollmann F (2018). Light-driven enzymatic decarboxylation of fatty acids. Angew Chem Int Ed Engl.

[9] Kretzschmar A, Otto C, Holz M, Werner S, Hübner L, Barth G (2013). Increased homologous integration frequency in *Yarrowia lipolytica* strains defective in non-homologous end-joining. Curr Genet.

[10] Verbeke J, Beopoulos A, Nicaud J-M (2013). Efficient homologous recombination with short length flanking fragments in Ku70 deficient *Yarrowia lipolytica* strains. Biotechnol Lett.

[11] Kunes S, Botstein D, Fox MS (1985). Transformation of yeast with linearized plasmid DNA. Formation of inverted dimers and recombinant plasmid products. J Mol Biol..

[12] Ledesma-Amaro R, Dulermo R, Niehus X, Nicaud J-M (2016). Combining metabolic engineering and process optimization to improve production and secretion of fatty acids. Metab Eng.

[13] Hirakawa K, Kobayashi S, Inoue T, Endoh-Yamagami S, Fukuda R, Ohta A (2009). Yas3p, an Opi1 family transcription factor, regulates cytochrome P450 expression in response to n-alkanes in *Yarrowia lipolytica*. J Biol Chem.

[14] Iida T, Sumita T, Ohta A, Takagi M (2000). The cytochrome P450ALK multigene family of an n-alkane-assimilating yeast, *Yarrowia lipolytica*: cloning and characterization of genes coding for new CYP52 family members. Yeast.

[15] Hennemann J, Iwasaki RS, Grund TN, Diensthuber RP, Richter F, Möglich A (2018). Optogenetic control by pulsed illumination. ChemBioChem.

[16] Hoenes K, Hess M, Vatter P, Spellerberg B, Hessling M (2018). 405 nm and 450 nm photoinactivation o*f Saccharomyces cerevisiae*. Eur J Microbiol Immunol..

[17] Robertson JB, Davis CR, Johnson CH (2013). Visible light alters yeast metabolic rhythms by inhibiting respiration. Proc Natl Acad Sci USA.

[18] Kim HM, Chae TU, Choi SY, Kim WJ, Lee SY (2019). Engineering of an oleaginous bacterium for the production of fatty acids and fuels. Nat Chem Biol.

[19] Mori K, Iwama R, Kobayashi S, Horiuchi H, Fukuda R, Ohta A (2013). Transcriptional repression by glycerol of genes involved in the assimilation of n-alkanes and fatty acids in yeast *Yarrowia lipolytica*. FEMS Yeast Res.

[20] Takai H, Iwama R, Kobayashi S, Horiuchi H, Fukuda R, Ohta A (2012). Construction and characterization of a *Yarrowia lipolytica* mutant lacking genes encoding cytochromes P450 subfamily 52. Fungal Genet Biol.

[21] Guadalupe-Medina V, Wouter Wisselink H, Luttik MAH, de Hulster E, Daran J-M, Pronk JT (2013). Carbon dioxide fixation by Calvin-Cycle enzymes improves ethanol yield in yeast. Biotechnol Biofuels..

[22] Li Y-J, Wang M-M, Chen Y-W, Wang M, Fan L-H, Tan T-W (2017). Engineered yeast with a CO_2_-fixation pathway to improve the bio-ethanol production from xylose-mixed sugars. Sci Rep.

[23] Xuan J-W, Fournier P, Gaillardin C (1988). Cloning of the LYS5 gene encoding saccharopine dehydrogenase from the yeast *Yarrowia lipolytica* by target integration. Curr Genet.

[24] Schwartz CM, Hussain MS, Blenner M, Wheeldon I (2016). Synthetic RNA Polymerase III promoters facilitate high-efficiency CRISPR-Cas9-mediated genome editing in *Yarrowia lipolytica*. ACS Synth Biol..

[25] Messerschmidt K, Hochrein L, Dehm D, Schulz K, Mueller-Roeber B (2016). Characterizing seamless ligation cloning extract for synthetic biological applications. Anal Biochem.

[26] Nadler F, Bracharz F, Kabisch J (2019). CopySwitch—in vivo optimization of gene copy numbers for heterologous gene expression in *Bacillus subtilis*. Front Bioeng Biotechnol..

[27] Labun K, Montague TG, Gagnon JA, Thyme SB, Valen E (2016). CHOPCHOP v2: a web tool for the next generation of CRISPR genome engineering. Nucleic Acids Res.

[28] Montague TG, Cruz JM, Gagnon JA, Church GM, Valen E (2014). CHOPCHOP: a CRISPR/Cas9 and TALEN web tool for genome editing. Nucleic Acids Res.

[29] Boeke JD, Trueheart J, Natsoulis G, Fink GR (1987). 5-Fluoroorotic acid as a selective agent in yeast molecular genetics. Methods Enzymol.

[30] Papanikolaou S, Aggelis G (2003). Modeling lipid accumulation and degradation in *Yarrowia lipolytica* cultivated on industrial fats. Curr Microbiol.

[31] Matthäus F, Ketelhot M, Gatter M, Barth G (2014). Production of lycopene in the non-carotenoid-producing yeast *Yarrowia lipolytica*. Appl Environ Microbiol.

[32] Zhou YJ, Buijs NA, Zhu Z, Gómez DO, Boonsombuti A, Siewers V (2016). Harnessing yeast peroxisomes for biosynthesis of fatty-acid-derived biofuels and chemicals with relieved side-pathway competition. J Am Chem Soc.

[33] Kang M-K, Nielsen J (2017). Biobased production of alkanes and alkenes through metabolic engineering of microorganisms. J Ind Microbiol Biotechnol.

[34] Cao Y-X, Xiao W-H, Zhang J-L, Xie Z-X, Ding M-Z, Yuan Y-J (2016). Heterologous biosynthesis and manipulation of alkanes in *Escherichia coli*. Metab Eng.

[35] Kang M-K, Zhou YJ, Buijs NA, Nielsen J (2017). Functional screening of aldehyde decarbonylases for long-chain alkane production by *Saccharomyces cerevisiae*. Microb Cell Fact.

[36] Chen B, Lee D-Y, Chang MW (2015). Combinatorial metabolic engineering of *Saccharomyces cerevisiae* for terminal alkene production. Metab Eng.

[37] Zhou YJ, Hu Y, Zhu Z, Siewers V, Nielsen J (2018). Engineering 1-alkene biosynthesis and secretion by dynamic regulation in yeast. ACS Synth Biol..

[38] Foo JL, Susanto AV, Keasling JD, Leong SSJ, Chang MW (2017). Whole-cell biocatalytic and *de novo* production of alkanes from free fatty acids in *Saccharomyces cerevisiae*. Biotechnol Bioeng.

