# Supplementary material for: Drop-in biofuel production using fatty acid photodecarboxylase from Chlorella variabilis in the oleaginous yeast Yarrowia lipolytica
Source: Biotechnol Biofuels. 2019 Aug 24;12:202. doi: 10.1186/s13068-019-1542-4 (PMC6708191; doi:10.1186/s13068-019-1542-4)
Supplement: Supplementary file 1 — Additional file 1: Seq. S1. Sequence of truncated CvFAP, optimized for Y. lipolytica codon usage. Table S1.Characterization of H222 Δku70, JMY5749, S33001. Table S2. Addendum for characterization of JMY5749 and S33001. Fig. S1. Intracellular fatty acid concentrations of Yarrowia JMY5749/CvFAP constructs and empty vector control. Table S3. Results of Anova method for coefficients shown in Fig. 4 A. Fig. S2. Impact of different light regimes on cultivations of JMY5749/CvFAP. Table S4. Results of Anova method for coefficients shown in Fig. 4B. Seq. S2. Sequence for DO-dependent automated feeding. Fig. S3. Comparison of CvFAP variant (S07004, S121F) and wild type (S07013), cultivated in triplicates. Fig. S4. Rendering of CvFAP WT and S121F variant. Fig. S5. Additional parameters of bioprocesses with four different light regimes (Fig. 5). Fig. S6. Preferred conversion of available substrates to hydrocarbons. Fig. S7. Vector maps of plasmids p15018 and p33001. Table S5. List of oligonucleotides. Table S6. List of constructed vectors. Fig. S8. Light emission of LED-device. Table S7. Correlation of light intensity and power supply values. Fig. S9. Hydrocarbon peaks vs background. [file 13068_2019_1542_MOESM1_ESM.docx]

### Additional file

**Drop-In Biofuel production by using fatty acid photodecarboxylase from *Chlorella variabilis* in the oleaginous yeast *Yarrowia lipolytica*.**

All figures, tables or sequences of this supplementary file are listed according to their appearance in the main section.

**Table of contents**

Seq. S1: Sequence of truncated CvFAP, optimized for *Y. lipolytica* codon usage. 1

Table S1: Characterization of H222 *Δku70*, JMY5749, S33001. 2

Table S2: Addendum for characterization of JMY5749 and S33001. 6

Fig. S1: Intracellular fatty acid concentrations of *Yarrowia* JMY5749/CvFAP constructs and empty vector control. 7

Table S3: Results of Anova method for coefficients shown in Fig. 4 A. 8

Fig. S2: Impact of different light regimes on cultivations of JMY5749/CvFAP. 9

Table S4: Results of Anova method for coefficients shown in Fig. 4 B. 12

Seq. S2: Sequence for DO-dependent automated feeding. 13

Fig. S3: Comparison of CvFAP variant (S07004, S121F) and wild type (S07013), cultivated in triplicates. 14

Fig. S4: Rendering of CvFAP WT and S121F variant. 18

Fig. S5: Additional parameters of bioprocesses with four different light regimes (Fig. 5). 19

Fig. S6: Preferred conversion of available substrates to hydrocarbons. 25

Fig. S7: Vector maps of plasmids p15018 and p33001. 26

Table S5: List of oligonucleotides. 27

Table S6: List of constructed vectors. 29

Fig. S8: Light emission of LED-device. 30

Table S7: Correlation of light intensity and power supply values. 31

Fig. S9 Hydrocarbon peaks vs background. 32

References 33

**Seq. S1: Sequence of truncated CvFAP, optimized for *Y. lipolytica* codon usage.**

The sequence was ordered from baseclear (B.V.).

ATGCGAGCCTCTGCTGTCGAGGACATTCGAAAGGTGCTCTCTGACTCCTCTTCCCCCGTCGCCGGACAGAAGTACGACTACATCCTGGTCGGAGGCGGTACCGCTGCTTGTGTGCTGGCTAACCGACTCTCCGCCGACGGCTCTAAGCGAGTCCTGGTGCTGGAGGCTGGTCCTGACAACACCTCCCGAGACGTGAAGATCCCCGCCGCTATTACCCGACTGTTCCGATCTCCCCTGGACTGGAACCTCTTCTCTGAGCTGCAGGAGCAGCTCGCTGAGCGACAAATCTACATGGCCCGAGGTCGACTGCTCGGAGGCTCTTCCGCCACCAACGCTACCCTGTACCACCGAGGCGCCGCTGGTGACTACGACGCTTGGGGAGTCGAGGGCTGGTCTTCCGAGGACGTCCTCTCCTGGTTCGTGCAGGCCGAGACCAACGCTGACTTCGGACCTGGTGCTTACCACGGCTCTGGTGGACCTATGCGAGTCGAGAACCCCCGATACACCAACAAGCAGCTGCACACCGCCTTCTTCAAGGCCGCTGAGGAAGTCGGACTCACCCCCAACTCCGACTTCAACGACTGGTCTCACGACCACGCTGGTTACGGAACCTTCCAGGTCATGCAGGACAAGGGCACCCGAGCCGACATGTACCGACAGTACCTGAAGCCCGTCCTCGGTCGACGAAACCTGCAGGTGCTCACCGGAGCCGCTGTCACCAAGGTGAACATTGACCAGGCTGCTGGAAAGGCTCAGGCTCTGGGCGTCGAGTTCTCCACCGACGGTCCCACCGGAGAGCGACTGTCCGCTGAGCTCGCTCCCGGCGGTGAGGTCATTATGTGTGCTGGTGCTGTGCACACCCCCTTCCTGCTCAAGCACTCTGGTGTGGGTCCTTCTGCTGAGCTGAAGGAGTTCGGTATCCCCGTCGTGTCCAACCTCGCTGGAGTGGGACAGAACCTGCAGGACCAGCCTGCTTGTCTCACCGCTGCTCCCGTGAAGGAGAAGTACGACGGAATCGCCATTTCTGACCACATCTACAACGAGAAGGGCCAGATTCGAAAGCGAGCCATCGCTTCCTACCTGCTCGGAGGCCGAGGTGGACTGACCTCTACCGGCTGTGACCGAGGTGCCTTCGTCCGAACCGCTGGTCAGGCTCTGCCCGACCTCCAGGTCCGATTCGTGCCTGGAATGGCTCTCGACCCCGACGGTGTCTCCACCTACGTGCGATTCGCCAAGTTCCAGTCCCAGGGTCTGAAGTGGCCCTCTGGAATTACCATGCAGCTCATCGCCTGTCGACCCCAGTCCACCGGCTCTGTGGGTCTGAAGTCTGCCGACCCCTTCGCCCCTCCCAAGCTCTCTCCTGGATACCTGACCGACAAGGACGGTGCCGACCTGGCTACCCTCCGAAAGGGTATTCACTGGGCTCGAGACGTGGCTCGATCTTCTGCTCTGTCCGAGTACCTCGACGGAGAGCTGTTCCCCGGTTCCGGAGTCGTGTCTGACGACCAGATCGACGAGTACATTCGACGATCTATCCACTCTTCCAACGCTATCACCGGTACCTGTAAGATGGGAAACGCCGGCGACTCTTCCTCTGTCGTGGACAACCAGCTGCGAGTCCACGGTGTGGAGGGACTCCGAGTCGTGGACGCCTCTGTCGTTCCTAAGATTCCCGGCGGTCAGACCGGAGCTCCTGTCGTGATGATTGCTGAGCGAGCCGCTGCCCTGCTCACTGGCAAGGCTACTATCGGTGCTTCCGCCGCCGCTCCTGCTACTGTGGCTGCTTAG

**Table S1: Characterization of H222 *Δku70*, JMY5749, S33001.**

Cells were transformed with CvFAP expression plasmid or empty vector control under two different light conditions (on/off, referred as blue/dark). Constructs were cultivated in lipid body formation inducing YSM medium (5 % glucose) under exposure of an readily available plant LED-light (for strain H222 *Δku70*) or a more distinct LED-strip (for strains JMY5749, S33001) for 96 h. Amounts of detected hydrocarbons per cell dry weight or culture volume and corresponding standard derivations are listed for each construct and condition.

| **Constructs** | **Light condition** | **Alkane/Alkene** | **Mean amount per cdw µg/mg** | **Standard deviation amount per cdw µg/mg** | **Mean amount per culture volume µg/L** | **Standard deviation amount per culture volume µg/L** |
| --- | --- | --- | --- | --- | --- | --- |
| H222 *Δku70* + CvFAP  (non- optimized extraction method) | blue | C15:1 | 0.000000 | 0.000000 | 0.0 | 0.0 |
| H222 *Δku70* + CvFAP  (non- optimized extraction method) | dark | C15:0 | 0.000000 | 0.000000 | 0.0 | 0.0 |
| H222 *Δku70* + CvFAP  (non- optimized extraction method) | dark | C15:1 | 0.000000 | 0.000000 | 0.0 | 0.0 |
| H222 *Δku70* + CvFAP  (non- optimized extraction method) | dark | C17:0 | 0.000000 | 0.000000 | 0.0 | 0.0 |
| H222 *Δku70* + CvFAP  (non- optimized extraction method) | dark | C17:2 | 0.000000 | 0.000000 | 0.0 | 0.0 |
| H222 *Δku70* + CvFAP  (non- optimized extraction method) | dark | C17:1 | 0.001268 | 0.000737 | 1.5 | 0.9 |
| H222 *Δku70* + CvFAP  (non- optimized extraction method) | blue | C15:0 | 0.010064 | 0.005915 | 10.9 | 7.1 |
| H222 *Δku70* + CvFAP  (non- optimized extraction method) | blue | C17:2 | 0.019164 | 0.009226 | 19.7 | 9.2 |
| H222 *Δku70* + CvFAP  (non- optimized extraction method) | blue | C17:1 | 0.031145 | 0.012653 | 31.9 | 13.0 |
| H222 *Δku70* + CvFAP  (non- optimized extraction method) | blue | C17:0 | 0.048694 | 0.010288 | 49.6 | 5.9 |
| H222 *Δku70* + empty vector control  (non- optimized extraction method) | blue | C15:0 | 0.000000 | 0.000000 | 0.0 | 0.0 |
| H222 *Δku70* + empty vector control  (non- optimized extraction method) | blue | C15:1 | 0.000000 | 0.000000 | 0.0 | 0.0 |
| H222 *Δku70* + empty vector control  (non- optimized extraction method) | blue | C17:0 | 0.000000 | 0.000000 | 0.0 | 0.0 |
| H222 *Δku70* + empty vector control  (non- optimized extraction method) | blue | C17:2 | 0.000000 | 0.000000 | 0.0 | 0.0 |
| H222 *Δku70* + empty vector control  (non- optimized extraction method) | dark | C15:0 | 0.000000 | 0.000000 | 0.0 | 0.0 |
| H222 *Δku70* + empty vector control  (non- optimized extraction method) | dark | C15:1 | 0.000000 | 0.000000 | 0.0 | 0.0 |
| H222 *Δku70* + empty vector control  (non- optimized extraction method) | dark | C17:0 | 0.000000 | 0.000000 | 0.0 | 0.0 |
| H222 *Δku70* + empty vector control  (non- optimized extraction method) | dark | C17:2 | 0.000000 | 0.000000 | 0.0 | 0.0 |
| H222 *Δku70* + empty vector control  (non- optimized extraction method) | blue | C17:1 | 0.001751 | 0.002891 | 1.5 | 2.4 |
| H222 *Δku70* + empty vector control  (non- optimized extraction method) | dark | C17:1 | 0.005416 | 0.007681 | 5.5 | 8.0 |
| JMY5749 + CvFAP  (non- optimized extraction method) | dark | C15:0 | 0.000000 | 0.000000 | 0.0 | 0.0 |
| JMY5749 + CvFAP  (non- optimized extraction method) | dark | C15:1 | 0.000000 | 0.000000 | 0.0 | 0.0 |
| JMY5749 + CvFAP  (non- optimized extraction method) | dark | C17:2 | 0.000746 | 0.000659 | 2.6 | 2.3 |
| JMY5749 + CvFAP  (non- optimized extraction method) | dark | C17:0 | 0.002857 | 0.001751 | 10.1 | 6.1 |
| JMY5749 + CvFAP  (non- optimized extraction method) | dark | C17:1 | 0.007524 | 0.002087 | 26.7 | 7.1 |
| JMY5749 + CvFAP  (non- optimized extraction method) | blue | C15:1 | 0.027968 | 0.008552 | 76.7 | 15.7 |
| JMY5749 + CvFAP(non- optimized extraction method) | blue | C15:0 | 0.113092 | 0.033040 | 310.4 | 58.9 |
| JMY5749 + CvFAP  (non- optimized extraction method) | blue | C17:2 | 0.132950 | 0.027600 | 368.5 | 47.6 |
| JMY5749 + CvFAP  (non- optimized extraction method) | blue | C17:0 | 0.136845 | 0.043067 | 375.4 | 81.9 |
| JMY5749 + CvFAP  (non- optimized extraction method) | blue | C17:1 | 0.151946 | 0.032313 | 420.4 | 50.6 |
| S33001 + CvFAP  (non- optimized extraction method) | blue | C15:0 | 0.000000 | 0.000000 | 0.0 | 0.0 |
| S33001 + CvFAP  (non- optimized extraction method) | blue | C15:1 | 0.000000 | 0.000000 | 0.0 | 0.0 |
| S33001 + CvFAP  (non- optimized extraction method) | dark | C15:0 | 0.000000 | 0.000000 | 0.0 | 0.0 |
| S33001 + CvFAP  (non- optimized extraction method) | dark | C15:1 | 0.000000 | 0.000000 | 0.0 | 0.0 |
| S33001 + CvFAP  (non- optimized extraction method) | dark | C17:0 | 0.000000 | 0.000000 | 0.0 | 0.0 |
| S33001 + CvFAP  (non- optimized extraction method) | dark | C17:2 | 0.000000 | 0.000000 | 0.0 | 0.0 |
| S33001 + CvFAP  (non- optimized extraction method) | dark | C17:1 | 0.001873 | 0.000055 | 6.7 | 0.4 |
| S33001 + CvFAP  (non- optimized extraction method) | blue | C17:0 | 0.003524 | 0.000326 | 11.7 | 0.8 |
| S33001 + CvFAP  (non- optimized extraction method) | blue | C17:2 | 0.006632 | 0.000795 | 22.1 | 2.3 |
| S33001 + CvFAP  (non- optimized extraction method) | blue | C17:1 | 0.006670 | 0.000617 | 22.2 | 1.4 |
| JMY5749 + empty vector control (non- optimized extraction method) | blue | C15:0 | 0.000000 | 0.000000 | 0.0 | 0.0 |
| JMY5749 + empty vector control (non- optimized extraction method) | blue | C15:1 | 0.000000 | 0.000000 | 0.0 | 0.0 |
| JMY5749 + empty vector control (non- optimized extraction method) | blue | C17:0 | 0.000000 | 0.000000 | 0.0 | 0.0 |
| JMY5749 + empty vector control (non- optimized extraction method) | blue | C17:1 | 0.000000 | 0.000000 | 0.0 | 0.0 |
| JMY5749 + empty vector control (non- optimized extraction method) | blue | C17:2 | 0.000000 | 0.000000 | 0.0 | 0.0 |
| JMY5749 + empty vector control (non- optimized extraction method) | dark | C15:0 | 0.000000 | 0.000000 | 0.0 | 0.0 |
| JMY5749 + empty vector control (non- optimized extraction method) | dark | C15:1 | 0.000000 | 0.000000 | 0.0 | 0.0 |
| JMY5749 + empty vector control (non- optimized extraction method) | dark | C17:0 | 0.000000 | 0.000000 | 0.0 | 0.0 |
| JMY5749 + empty vector control (non- optimized extraction method) | dark | C17:2 | 0.000000 | 0.000000 | 0.0 | 0.0 |
| JMY5749 + empty vector control (non- optimized extraction method) | dark | C17:1 | 0.000031 | 0.000049 | 0.1 | 0.2 |
| H222 *Δalk1* + empty vector control  (non- optimized extraction method) | blue | C15:0 | 0.000000 | 0.000000 | 0.0 | 0.0 |
| H222 *Δalk1* + empty vector control  (non- optimized extraction method) | blue | C15:1 | 0.000000 | 0.000000 | 0.0 | 0.0 |
| H222 *Δalk1* + empty vector control  (non- optimized extraction method) | blue | C17:0 | 0.000000 | 0.000000 | 0.0 | 0.0 |
| H222 *Δalk1* + empty vector control  (non- optimized extraction method) | blue | C17:1 | 0.000000 | 0.000000 | 0.0 | 0.0 |
| H222 *Δalk1* + empty vector control  (non- optimized extraction method) | blue | C17:2 | 0.000000 | 0.000000 | 0.0 | 0.0 |
| H222 *Δalk1* + empty vector control  (non- optimized extraction method) | dark | C15:0 | 0.000000 | 0.000000 | 0.0 | 0.0 |
| H222 *Δalk1* + empty vector control  (non- optimized extraction method) | dark | C15:1 | 0.000000 | 0.000000 | 0.0 | 0.0 |
| H222 *Δalk1* + empty vector control  (non-optimized extraction method) | dark | C17:0 | 0.000000 | 0.000000 | 0.0 | 0.0 |
| H222 *Δalk1* + empty vector control  (non-optimized extraction method) | dark | C17:2 | 0.000000 | 0.000000 | 0.0 | 0.0 |
| H222 *Δalk1* + empty vector control  (non-optimized extraction method) | dark | C17:1 | 0.000168 | 0.000174 | 0.6 | 0.6 |

**Table S2: Addendum for characterization of JMY5749 and S33001, harbouring CvFAP expression plasmid or empty vector control** (listed in Tab. S1), regarding cell dry weight, consumed glucose in g/L and corresponding standard derivations.

| **Variant** | **Light** | **Mean cdw g/mL** | **Standard deviation cdw g/mL** | **Mean consumed glucose g/L** | **Standard deviation consumed glucose g/L** |
| --- | --- | --- | --- | --- | --- |
| JMY5749 + CvFAP  (non-optimized extraction method) | blue | 0.0036 | 0.0005 | 15.6 | 11.1 |
| JMY5749 + CvFAP  (non- optimized extraction method) | dark | 0.0040 | 0.0002 | 19.4 | 2.3 |
| S33001 + CvFAP  (non- optimized extraction method) | blue | 0.0040 | 0.0005 | 16.3 | 5.6 |
| S33001 + CvFAP  (non- optimized extraction method) | dark | 0.0044 | 0.0008 | 20.9 | 5.9 |
| JMY5749 + empty vector control (non- optimized extraction method) | blue | 0.0049 | 0.0006 | 10.6 | 5.0 |
| JMY5749 + empty vector control (non- optimized extraction method) | dark | 0.0047 | 0.0007 | 13.3 | 3.8 |
| S33001 + empty vector control  (non- optimized extraction method) | blue | 0.0034 | 0.0004 | 23.8 | 2.4 |
| S33001 + empty vector control  (non- optimized extraction method) | dark | 0.0069 | 0.0016 | 11.4 | 11.8 |

**Fig. S1: Intracellular fatty acid concentrations of *Yarrowia* JMY5749/CvFAP constructs and empty vector control**.

Cultivation conditions and description of lipid analytics are listed in material and method section. The concentration of each fatty acid is indicated by dots (in triplicates), the sum (medium) of fatty acids and the fatty acid composition is represented by a bar plot. Total fatty acid concentration was significant lower (Students two Sample t-test: t=3.2778, df=4, p-value=0.03056) for JMY5749/CvFAP construct (21.20272+/-4.396019) in comparison to empty vector control (35.08924+/-5.875347).


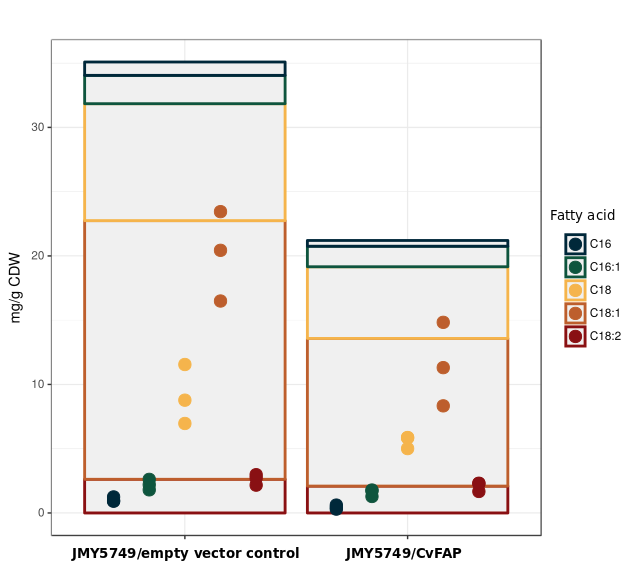


**Table S3: Results of Anova method** **for coefficients shown in Fig. 4 A**.

For calculations, R/RStudio were used as described in material and method section.

| **Coefficients** | **Estimate** | **Std. Error** | **t value** | **Pr(>\|t\|)** | **Signif. code** |
| --- | --- | --- | --- | --- | --- |
| (Intercept) | -5.426e-19 | 2.935e-04 | 0.000 | 1.0000 |  |
| ConditionFull-Intensity & Pulse 0ms | 9.805e-03 | 5.359e-04 | 18.296 | 3.76e-12 | *** |
| ConditionFull-Intensity & Pulse 100ms | 6.939e-03 | 5.359e-04 | 12.949 | 6.78e-10 | *** |
| ConditionFull-Intensity & Pulse 5000ms | 1.881e-03 | 5.359e-04 | 3.511 | 0.0029 | ** |
| ConditionHalf-Intensity & Pulse 0ms | 7.898e-03 | 5.359e-04 | 14.738 | 9.95e-11 | *** |
| ConditionHalf-Intensity & Pulse 100ms | 7.100e-03 | 6.227e-04 | 11.403 | 4.29e-09 | *** |
| ConditionHalf-Intensity & Pulse 5000ms | 9.154e-04 | 6.227e-04 | 1.470 | 0.1609 |  |

**Fig. S2: Impact of different light regimes on cultivations of** **JMY5749/CvFAP.**

**A:** Time-resolved logarithmized optical density measurements (corresponding light biotransformations shown in Fig. 4) were performed using a plate reader (PHERAstar FSX, BMG Labtech, Germany).


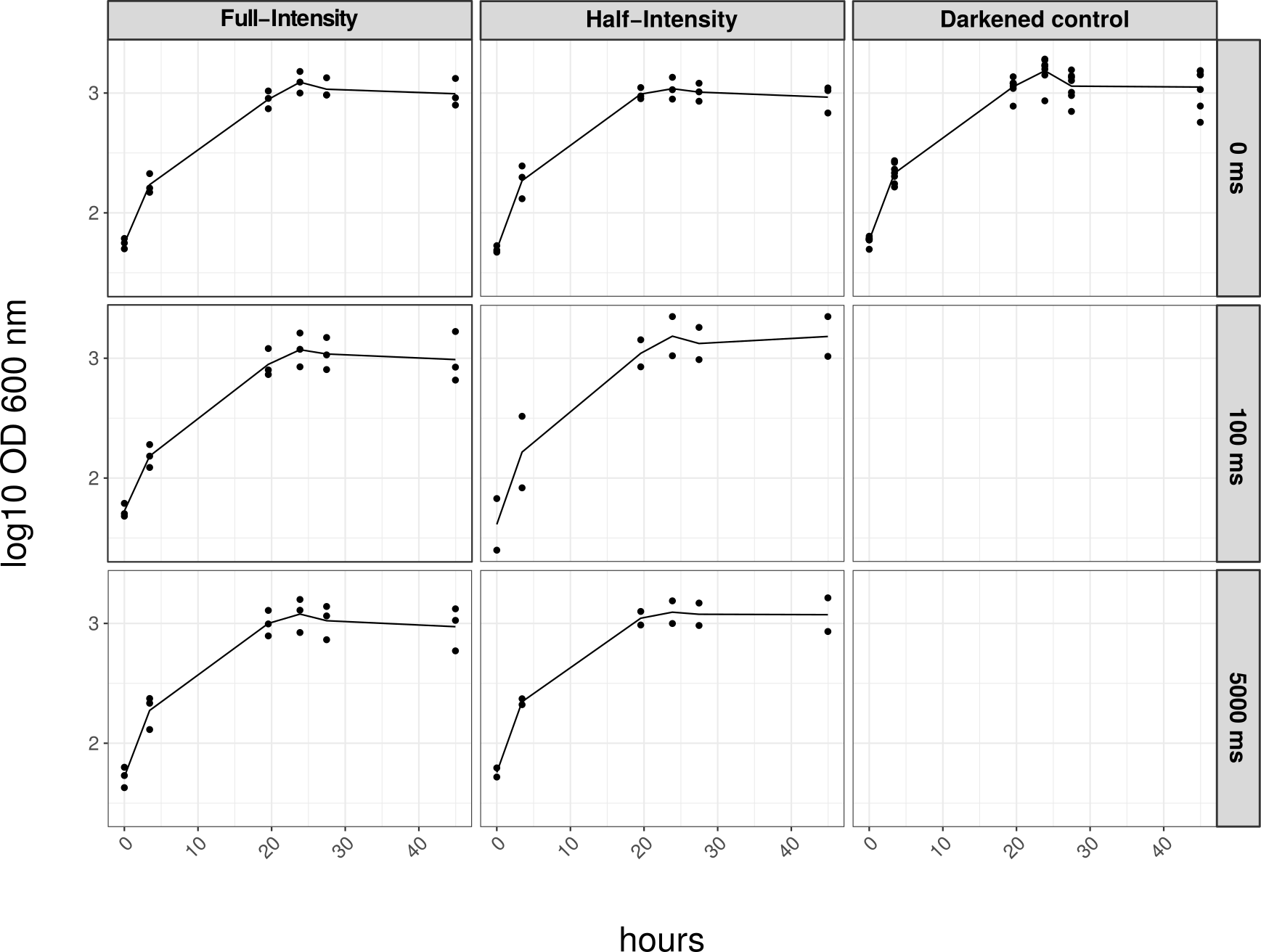


**Fig. S2 Impact of different light regimes on cultivations of** **JMY5749/CvFAP.**

**B:** In opposite to high cell density biotransformation (A), initial OD_600_ were set to 0.1 to determine the effect of different light intensities on cell growth. Full light intensity was determined as 28.7-32.3 µmol quanta m^-2^ s^-1^ per well. Light regimes were tested in triplicates, except for half intensity, pulse 100 and 5000 ms, which were cultivated in duplicates.


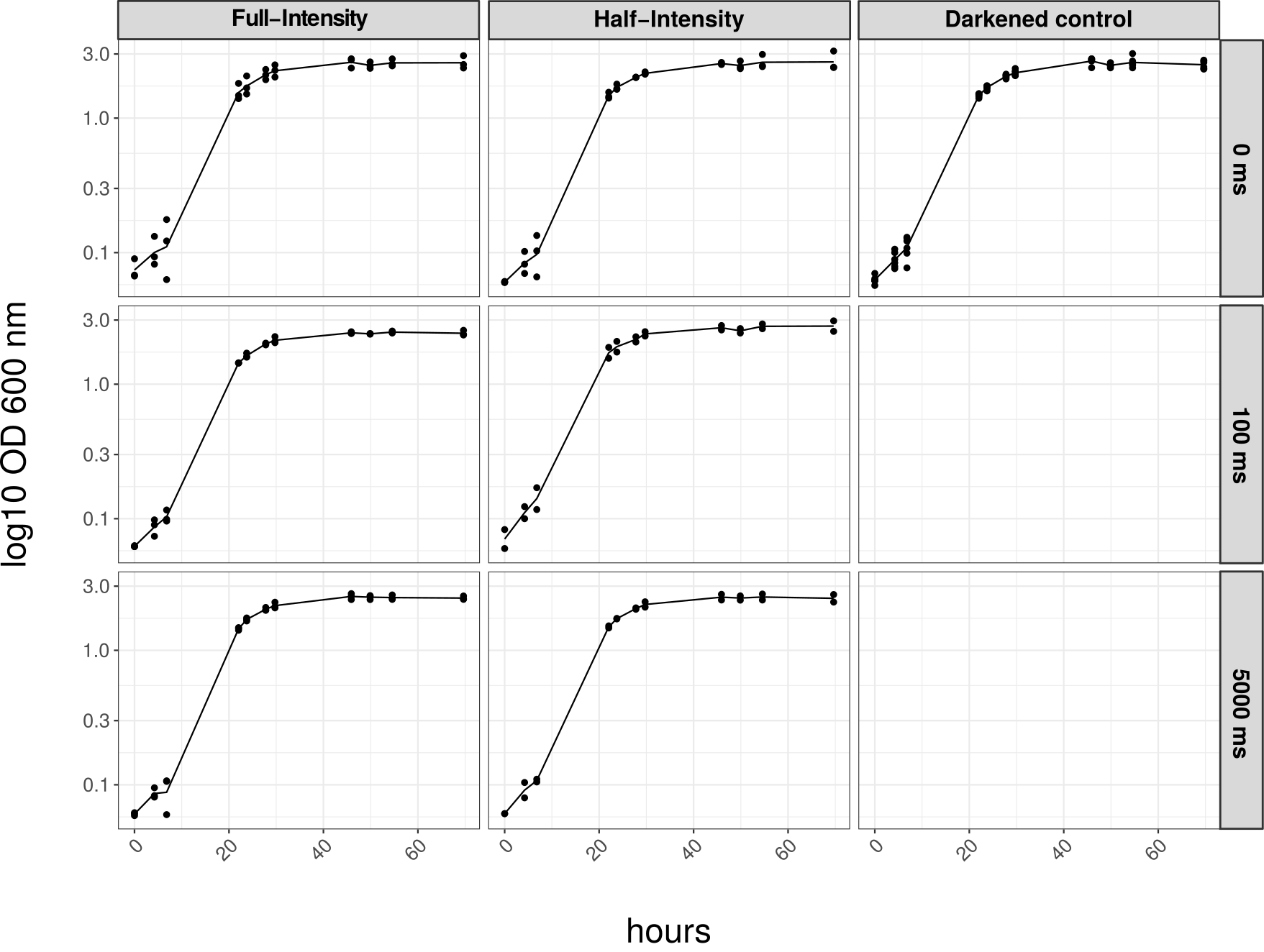


**Fig. S2 Impact of different light regimes on cultivations of** **JMY5749/CvFAP.**

**C:** Endpoint measurements of total hydrocarbon formation for cultivations (initial OD_600_ 0.1) of different clones, harbouring the genomic integration of *Cv*FAP cassette. Strains were cultivated in triplicates, expect for S07013. Hydrocarbon composition for endpoint measurements of S07004 and S07013 are shown in Figure 4 B.

**
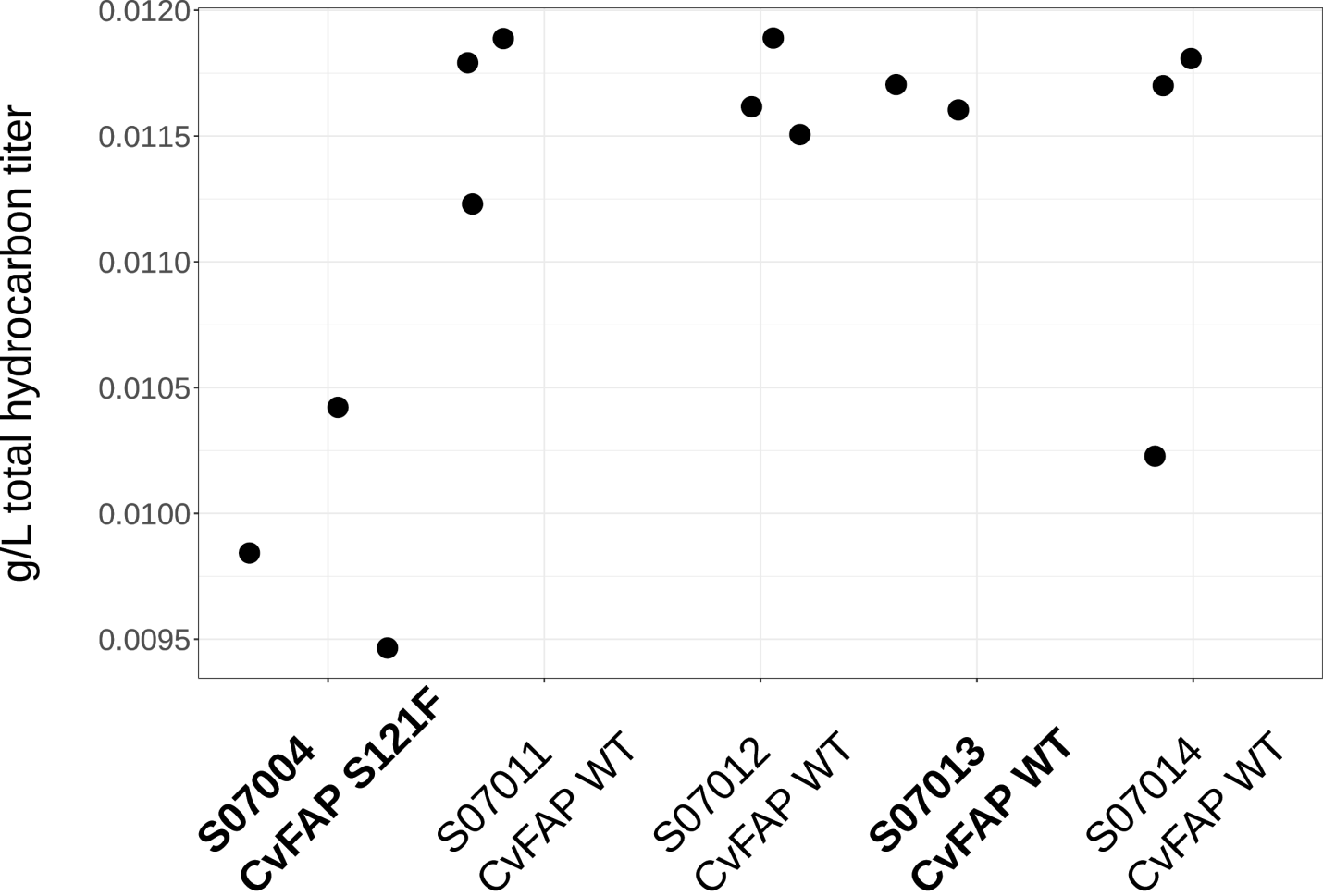
**

**Table S4: Results of Anova method** **for coefficients shown in Fig. 4 B**.

For calculations, R/RStudio were used as described in material and method section.

| **Coefficients** | **Estimate** | **Std. Error** | **t value** | **Pr(>\|t\|)** | **Signif. code** |
| --- | --- | --- | --- | --- | --- |
| (Intercept) | 0.0001653 | 0.0004361 | 0.379 | 0.7100 |  |
| ConditionFull-Intensity & Pulse 0ms | 0.0087410 | 0.0007553 | 11.573 | 7.08e-09 | *** |
| ConditionFull-Intensity & Pulse 100ms | 0.0080151 | 0.0007553 | 10.612 | 2.27e-08 | *** |
| ConditionFull-Intensity & Pulse 5000ms | 0.0018260 | 0.0007553 | 2.418 | 0.0288 | * |
| ConditionHalf-Intensity & Pulse 0ms | 0.0085443 | 0.0007553 | 11.313 | 9.63e-09 | *** |
| ConditionHalf-Intensity & Pulse 100ms | 0.0053621 | 0.0008721 | 6.149 | 1.86e-05 | *** |
| ConditionHalf-Intensity & Pulse 5000ms | 0.0009589 | 0.0008721 | 1.100 | 0.2889 |  |

**Seq. S2: Sequence for DO-dependent automated feeding.**

The sequence was developed by using software IRIS v6.0 (6.0.1054.817) from Infors HT, Switzerland.

#0, DO dropping,10

IF(pO2.v<40){SEQ=1}

#1,Check feeding volume,1

IF(Feed_Pump.v>100){Feed.sp=0 AND SEQ=0}ELSE{SEQ=2}

#2,DO Check,10

IF(pO2.v>70){Feed.sp=100 AND SEQ=3}ELSE{Feed.sp=0}

#3,Feeding,1

IF(SEQ_TIME>XXX dependent of pump performance XXX){Feed.sp=0;SEQ=4}

#4,Pause,10

IF(SEQ_TIME>Time(1:0)){SEQ=1}

**Fig. S3: Comparison of CvFAP variant (S07004, S121F) and wild type (S07013), cultivated in triplicates.**

**A:** Time-resolved DO concentrations as a representation of metabolic activity. Light intensity (height) and exposure time (width) are indicated by the blue shaded areas (light intensity was set to ~60 µm quanta m^-2^ s^-1^ which is ~1/10th of full intensity).

**
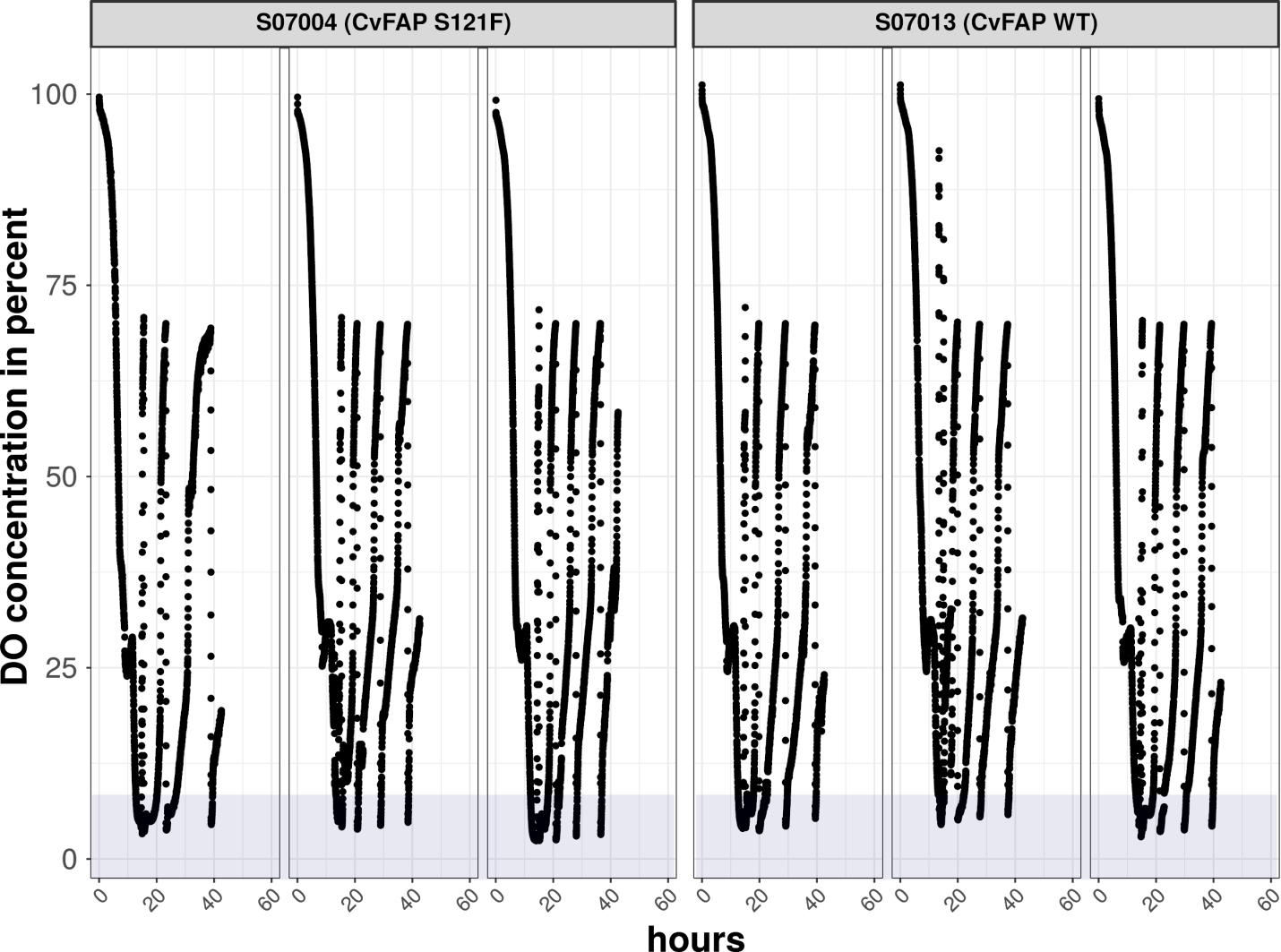
**

**Fig. S3: Comparison of CvFAP variant (S07004, S121F) and wild type (S07013), cultivated in triplicates.**

**B:** Time-resolved measurements of cell dry weights (cdw), to determine biomass accumulation in mg/ml.

**
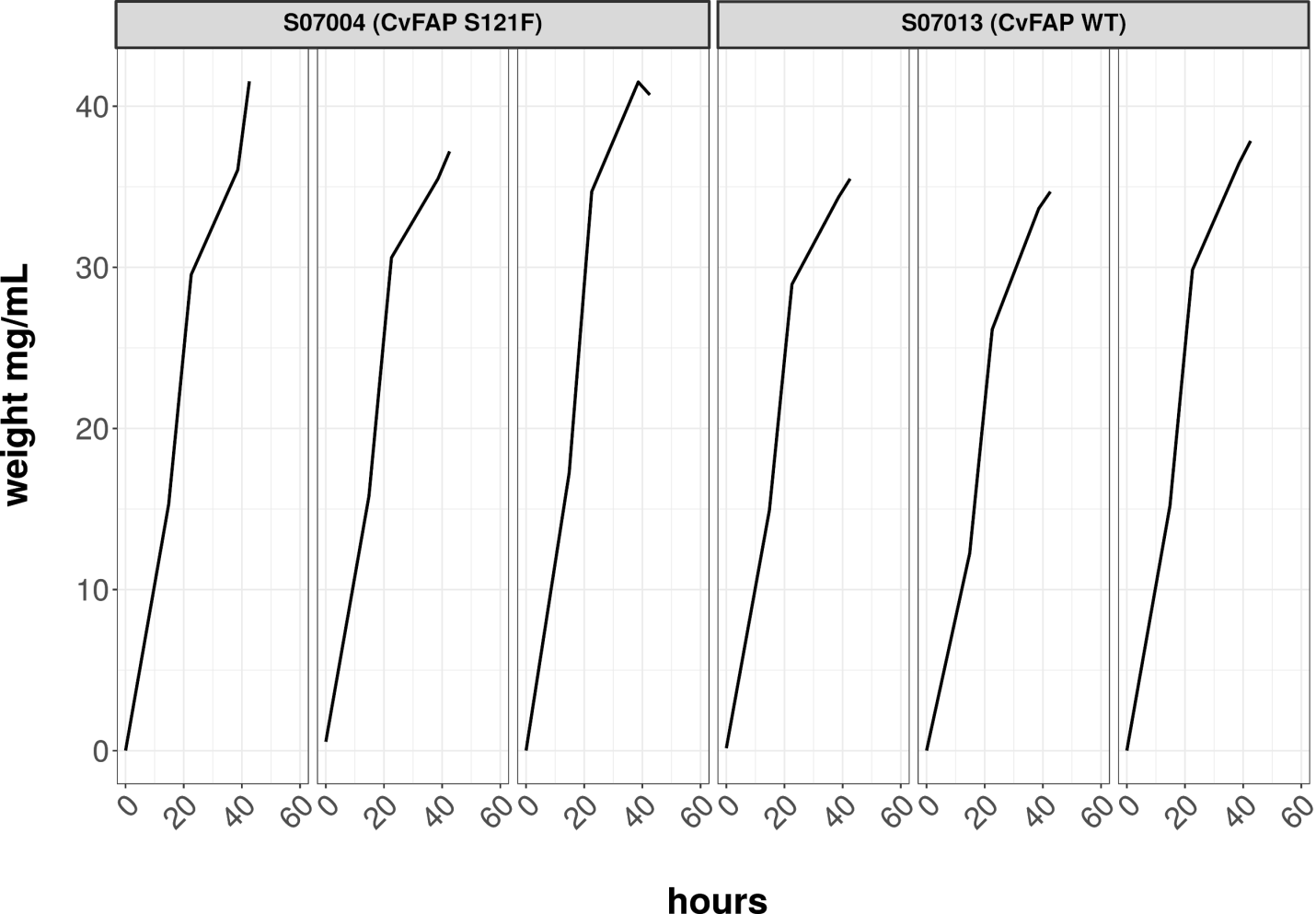
**

**Fig. S3: Comparison of CvFAP variant (S07004, S121F) and wild type (S07013), cultivated in triplicates.**

**C:** Time-resolved measurements of total intracellular hydrocarbon titer in g/L.


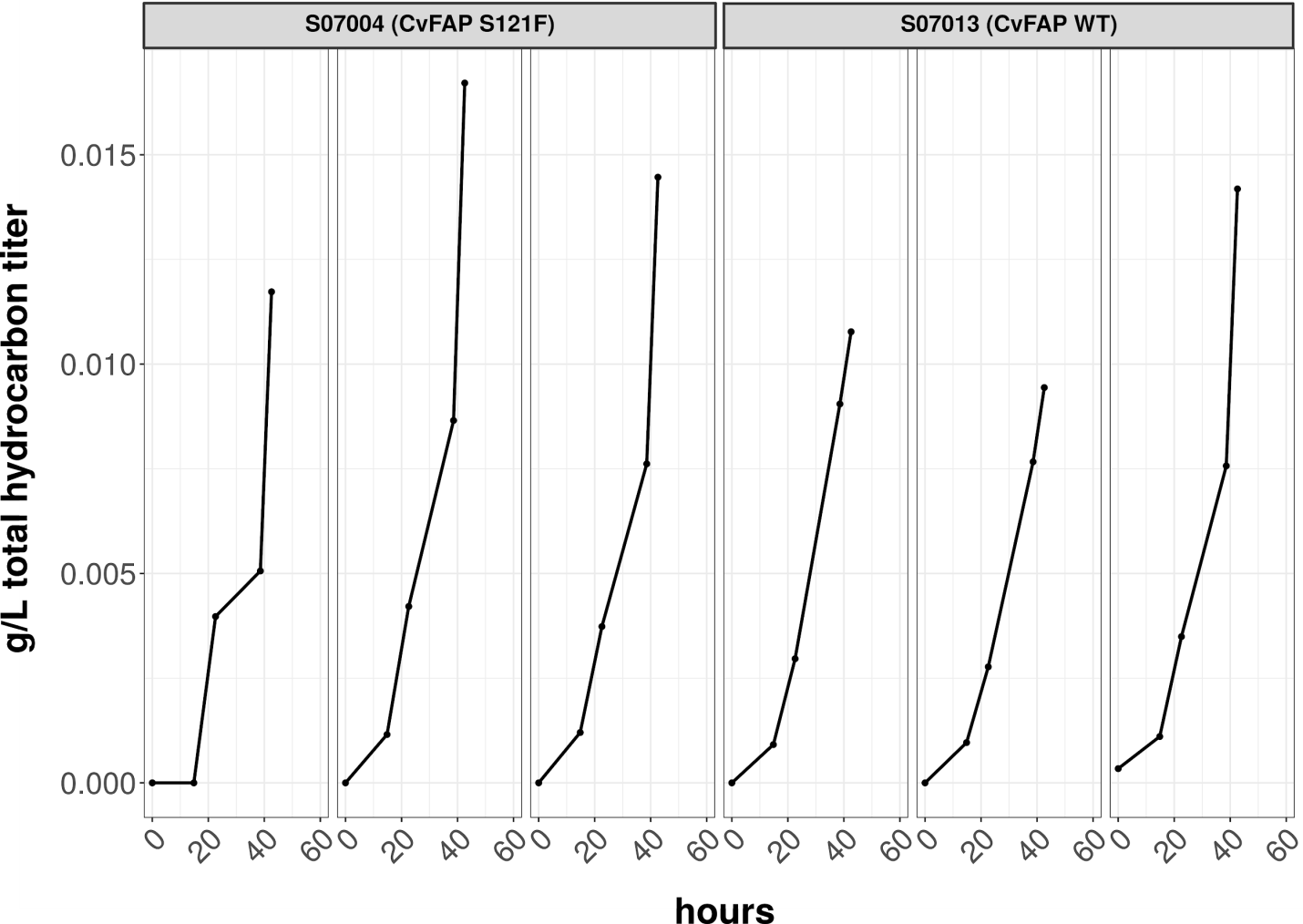


**Fig. S3: Comparison of CvFap variant (S07004, S121F) and wild type (S07013), cultivated in triplicates.**

**D:** Time-resolved distribution of measured hydrocarbons in percent.

**
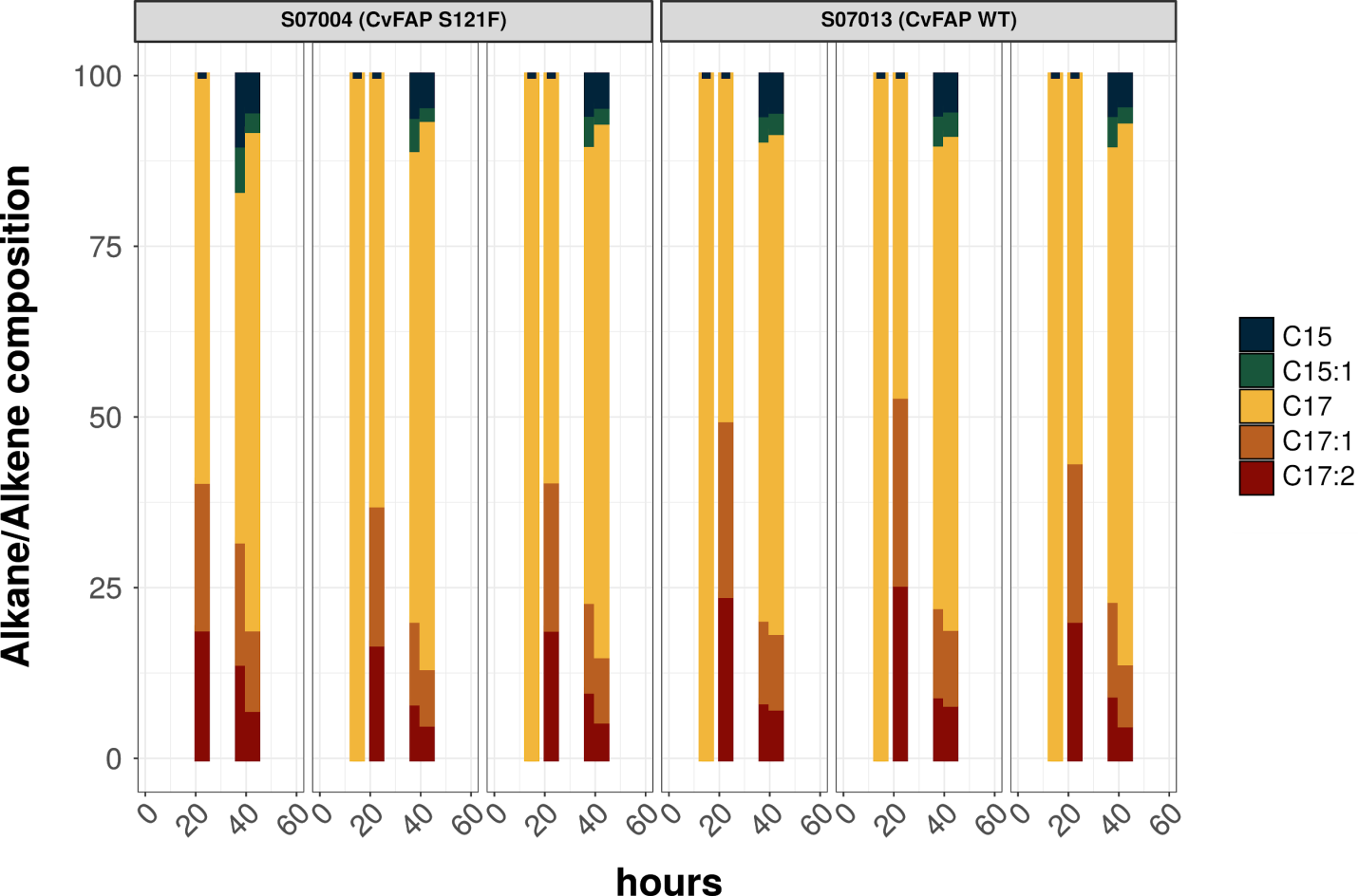
**

**Fig. S4: Rendering of CvFAP WT and S121F variant.**

For the rendering of the enzymes 3D structure, PyMOL 1.7.x was used. The CvFAP WT variant is shown in blue, while the S121F mutant is coloured red. A homology model of the mutant was created by using phyre2 algorithm [1] and pdb structure of the wild type (5NCC), deposited by [2]. In this overview of the cofactor binding side, the minimum distance between AA 121 phenylalanine ring and flavin adenine dinucleotide was calculated as 11.7 Ångström.


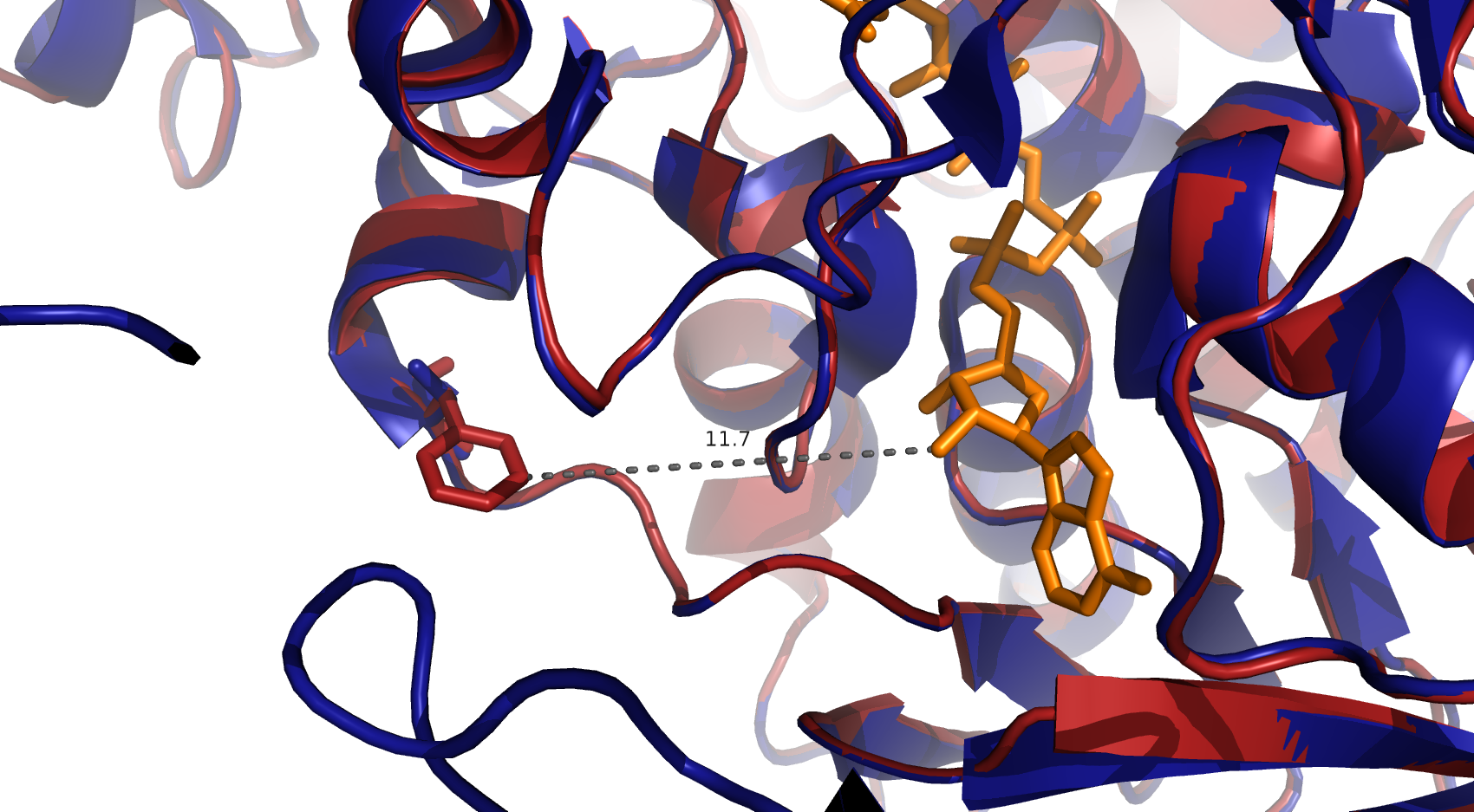


**Fig. S5: Additional parameters of bioprocesses with four different light regimes (Fig. 5).**

Bioprocesses with four different light regimes were characterized in triplicates by cultivation of strain S07004 as shown in the main section.

**A1**: Time-resolved fatty acid compositions listed for cultivation (in fermenter A-F) and light intensity (A-C: Full light intensity, D-F: No blue light control). Composition of fatty acids are coloured according to the legend. Within the first hours of cultivation not all fatty acid moieties were detectable.


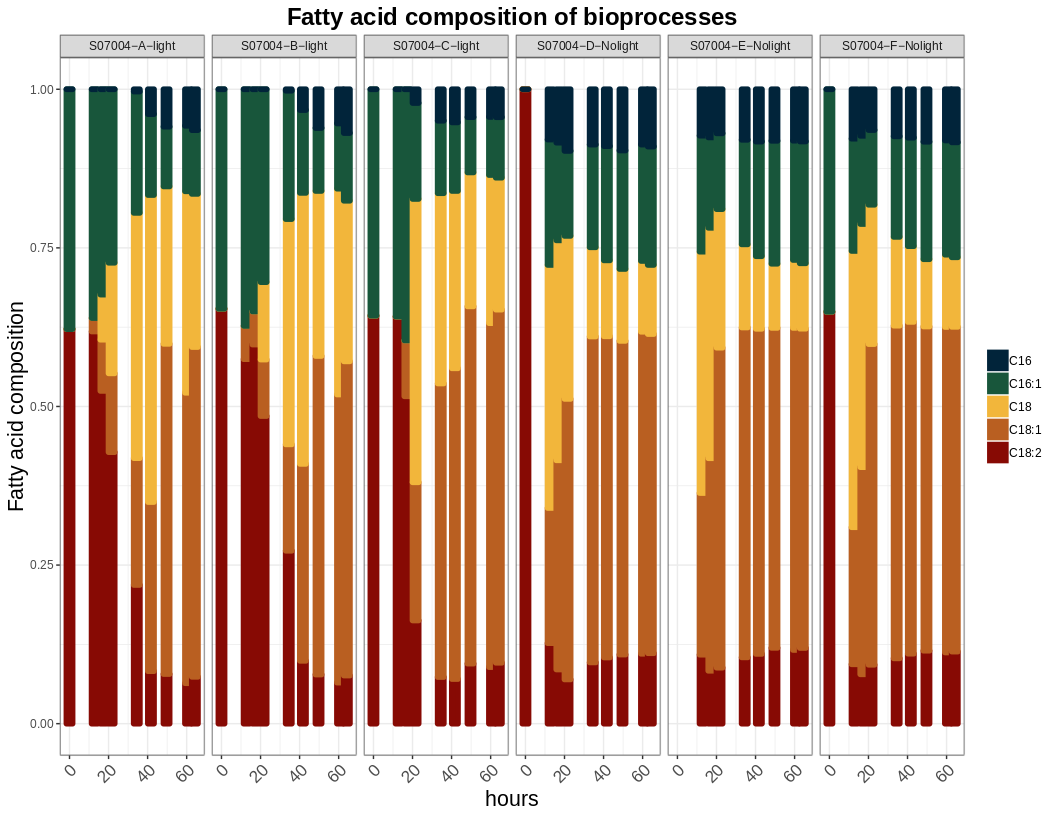


**Fig. S5: Additional parameters of bioprocesses with four different light regimes (Fig. 5).**

**A2:** Time-resolved fatty acid compositions listed for cultivation (in fermenter A-F) and light intensity (A-C: Half intensity, D-F: Light induction). Composition of fatty acids are coloured according to the legend.


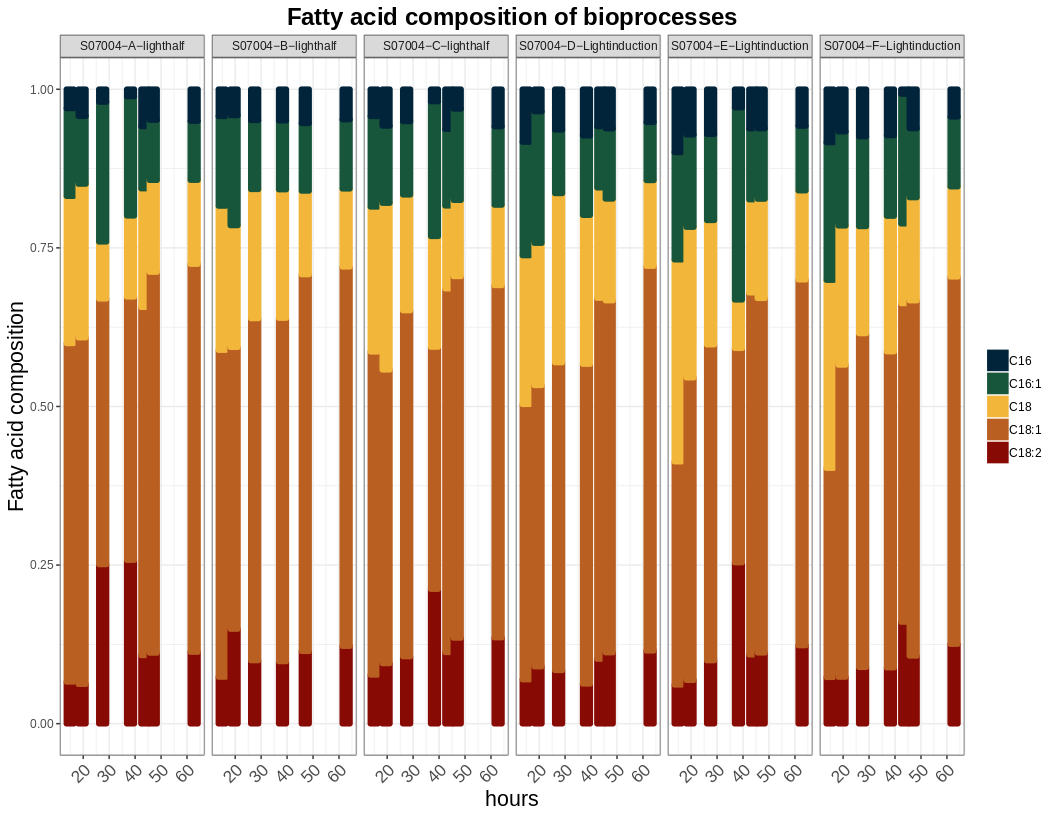


**Fig. S5: Additional parameters of bioprocesses with four different light regimes (Fig. 5).**

**B1:** Time-resolved titer of expected extracellular metabolites listed for fermentation (A-F) and light intensity (A-C: Full light intensity, D-F: No blue light control). Titers of the polyols D-arabitol, meso-erythritol and D-mannitol, the substrate glycerol and by-product citric acid are coloured according to the legend.

**
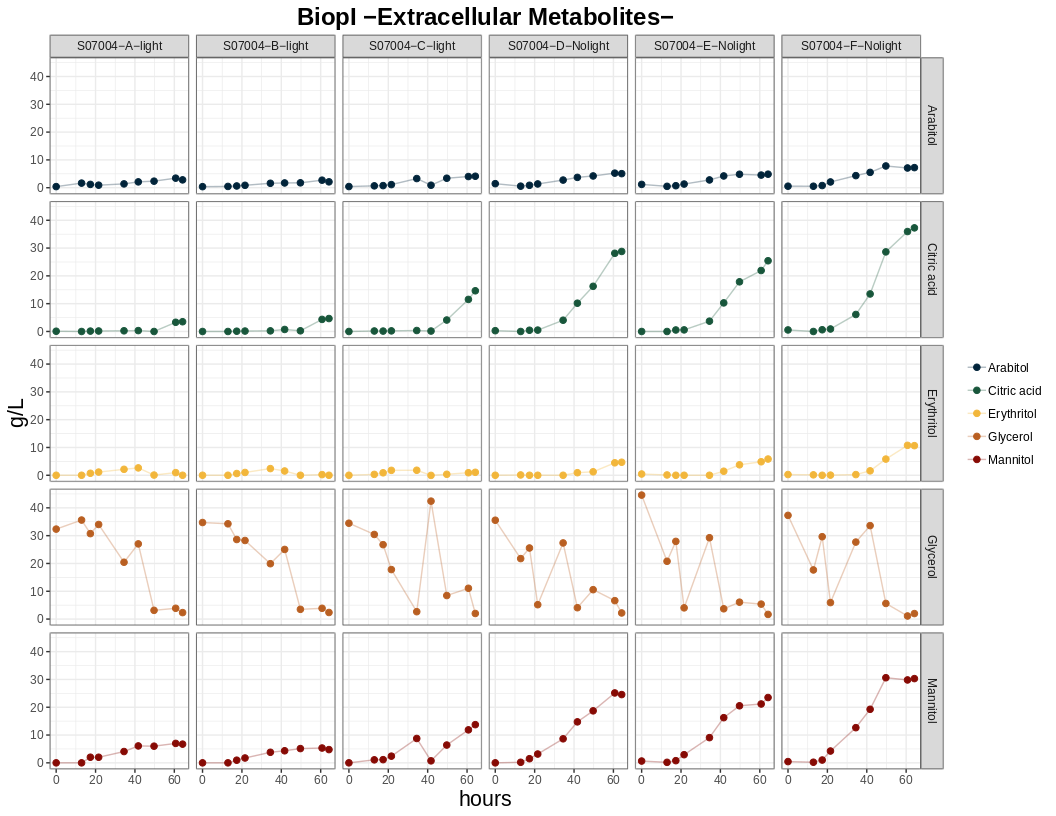
**

**Fig. S5: Additional parameters of bioprocesses with four different light regimes (Fig. 5).**

**B2:** Time-resolved titer of expected extracellular metabolites listed for fermentation (A-F) and light intensity (A-C: Half intensity, D-F: Light induction). Titers of the polyols D-arabitol, meso-erythritol and D-mannitol, the substrate glycerol and by-product citric acid are coloured according to the legend.

**
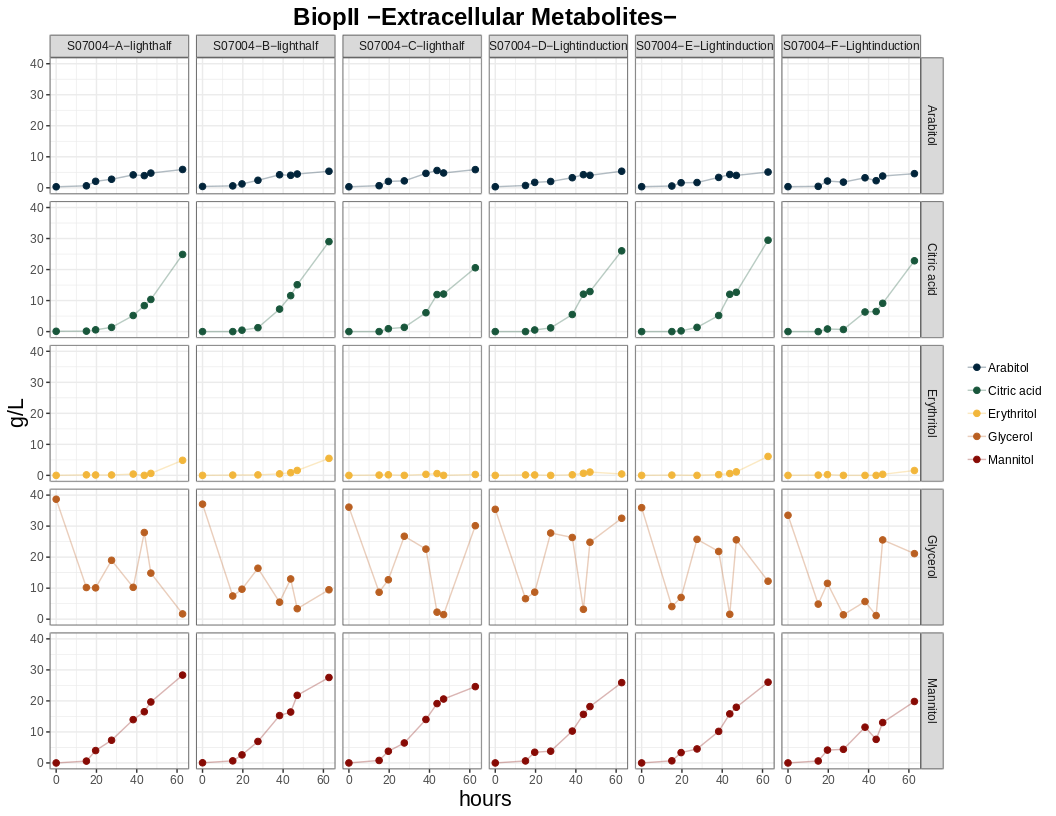
**

**Fig. S5: Additional parameters of bioprocesses with four different light regimes (Fig. 5).**

**C1**: Time-resolved hydrocarbon compositions listed for cultivation (in fermenter A-F) and light intensity (A-C: Full light intensity, D-F: No blue light control). Composition of alkanes and alkenes are coloured according to the legend.


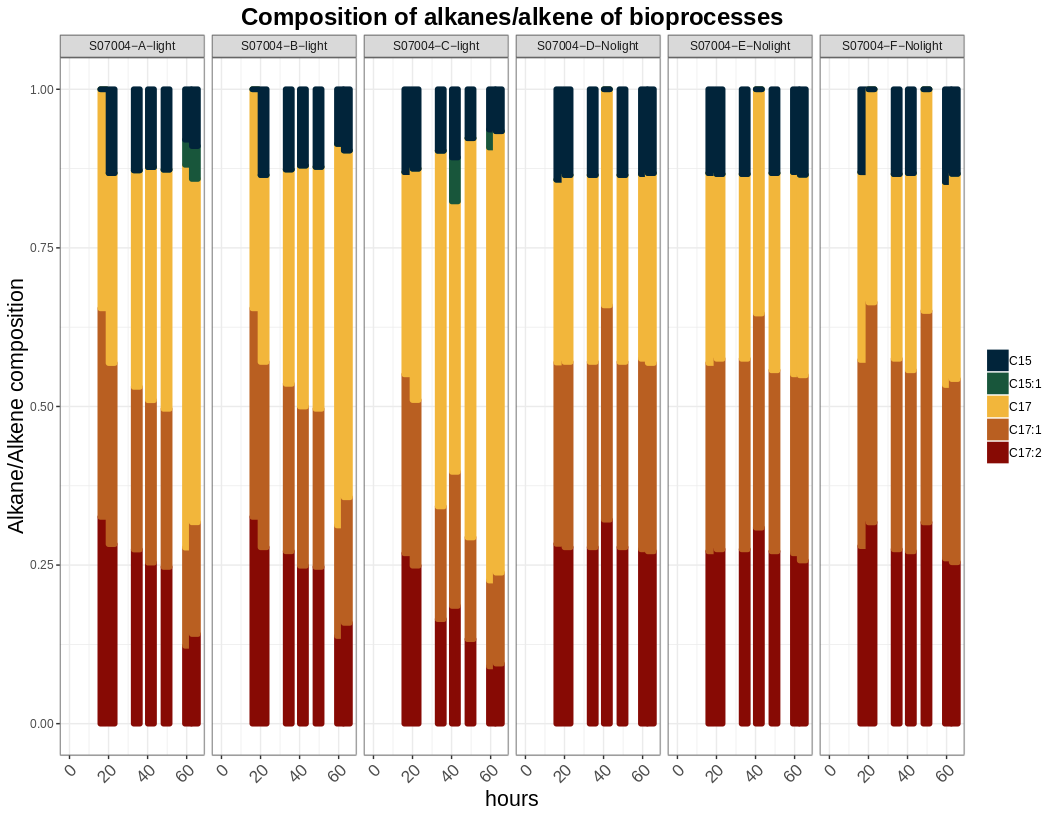


**Fig. S5: Additional parameters of bioprocesses with four different light regimes (Fig. 5).**

**C2:** Time-resolved hydrocarbon compositions listed for cultivation (in fermenter A-F) and light intensity (A-C: Half intensity, D-F: Light induction). Composition of alkanes and alkenes are coloured according to the legend.


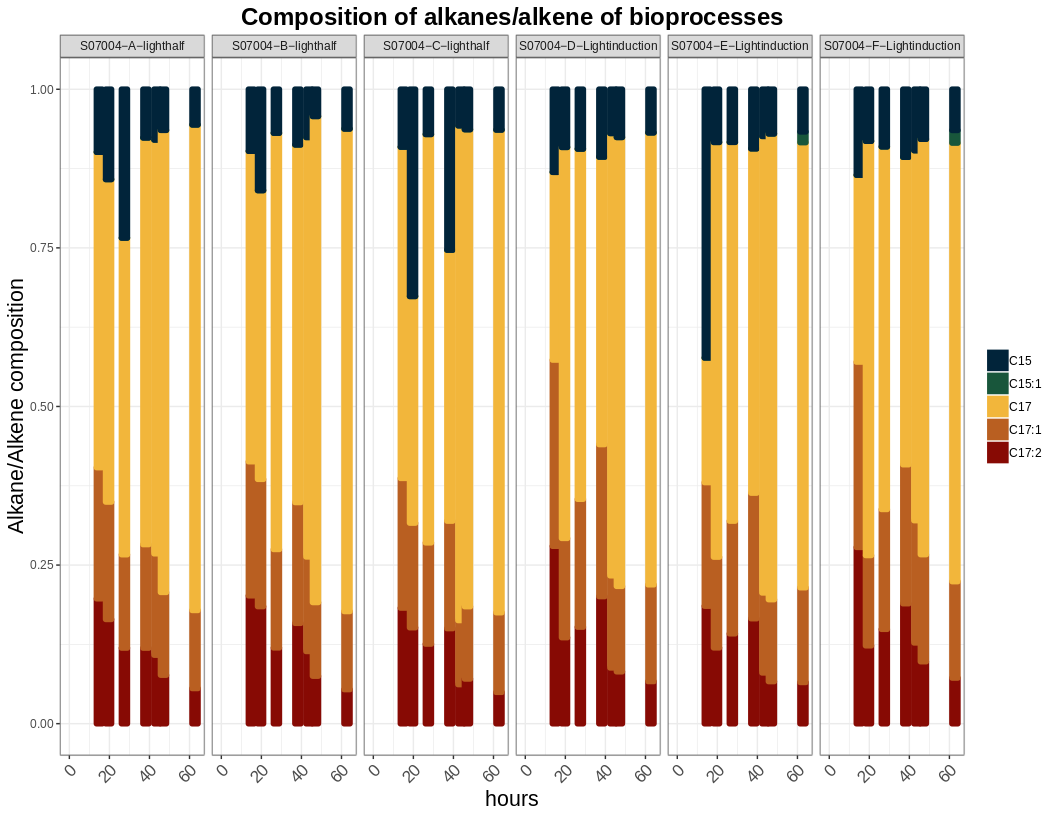


**Fig. S6: Preferred conversion of available substrates to hydrocarbons.**

Time resolved amounts of fatty acids and hydrocarbons present within the cultivation period. Axis are scaled logarithmic (base 10), one dot depict the intracellular fatty acid titer (substrate) and the corresponding hydrocarbon titer (converted product) of a single sample.

**
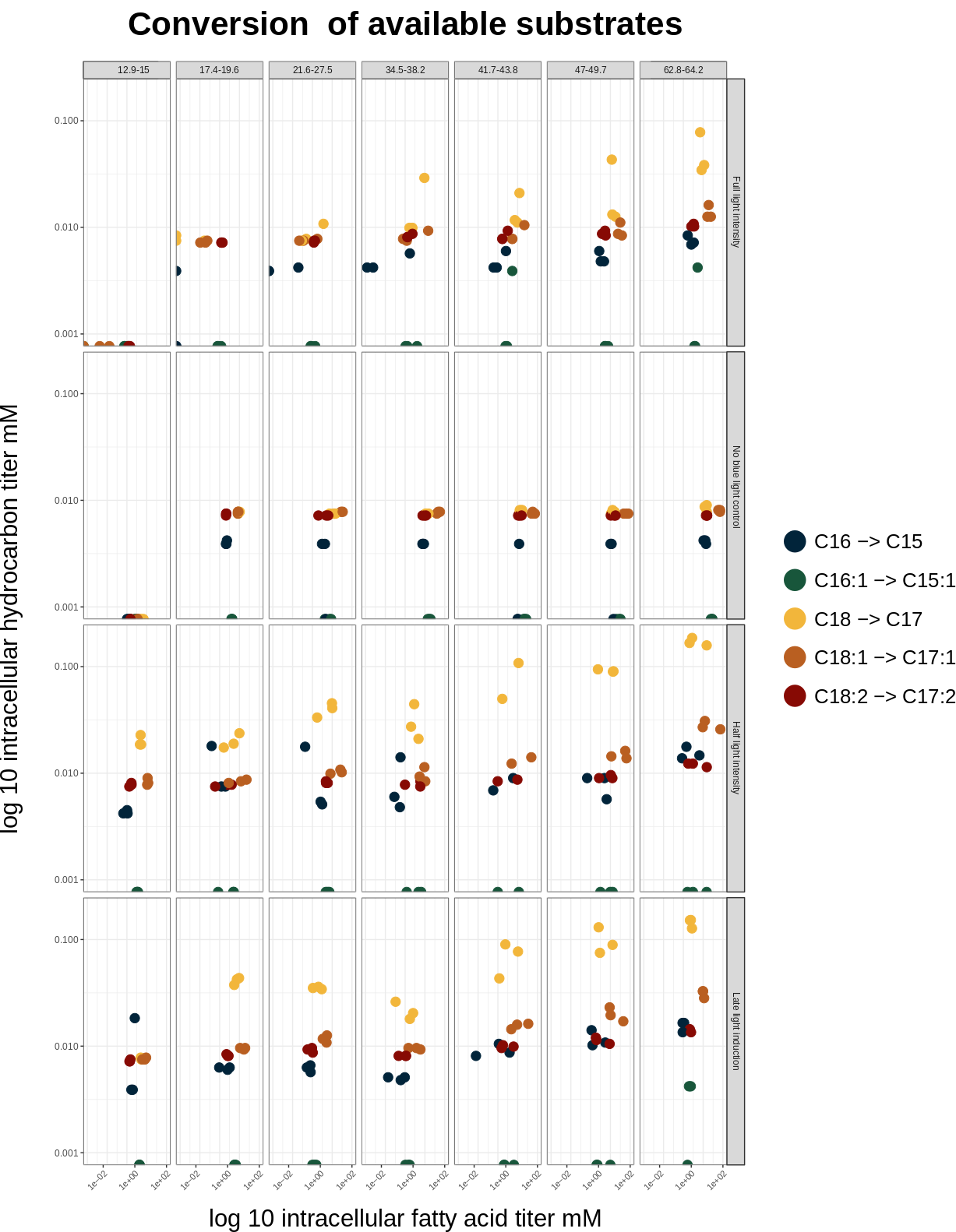
**

**Fig. S7: Vector maps of plasmids p15018 and p33001.**

Vector maps were created by using Geneious v.10.2.3.

**A:** The replicative, centromeric *Y. lipolytica, E. coli* shuttle-plasmid p15018 consists of CEN1-2, ARS68 region, pUC1 ori, kanamycin, ampicillin resistance cassette, as well as native *pTEF1* promoter and *tXPR2* terminator for gene expression and hphNT2 cassette (HPH) encoding for hygromycin resistance.
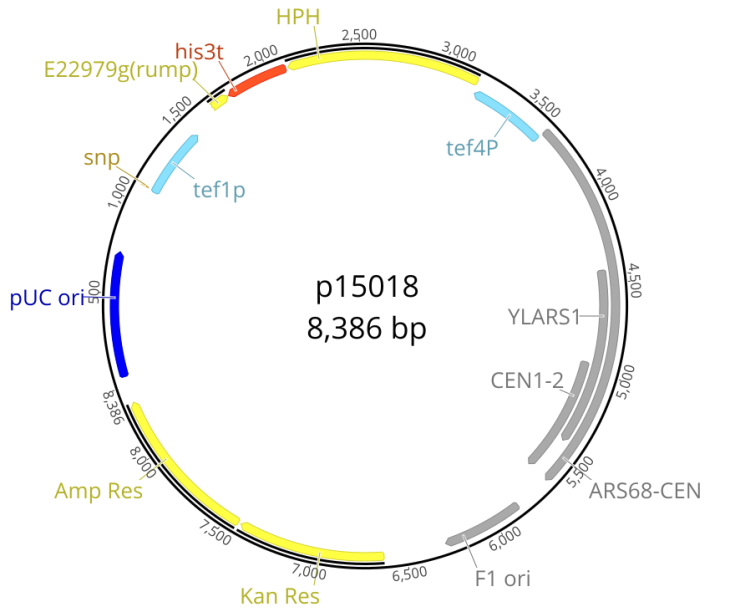


**B:** The replicative, centromeric *S.cerevisiae, E. coli* shuttle-plasmid p33001 serves as integration vector in *Y.lipolytica*. It consists of 2µ region, *ScURA3* marker gene, pBR322 origin and ampR marker. Front and Back region are homologous to promoter and terminator region of *YlALK1*.
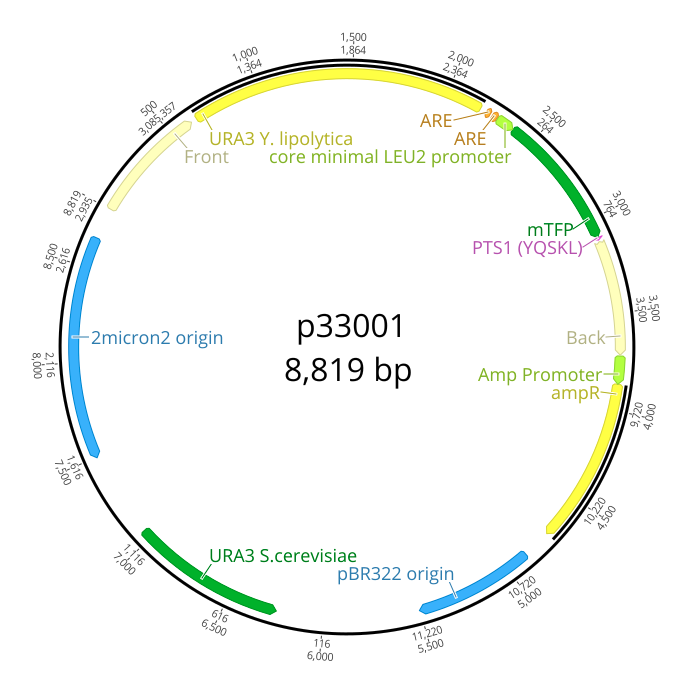


**Table** **S5: List of oligonucleotides.**

Primers were purchased from Eurofins Scientific, Luxembourg.

| **Designation** | **Sequence** | **Description** |
| --- | --- | --- |
| o13001 | GGAATAAGGGCGACACGGAA | For sequencing of corresonding sgRNA using pCRISPRyl or derived plasmids |
| o13002 | TACTTGCTTGAATATACAGTAGTATGCGGCCGCTTCTACAATAGCTTTATTGGCCCTATTGAG | Fwd oligo for amplification of CvFAP/hphNT1 cassette with overlapping homologous region to p15018 |
| o13003 | GAGCTGCATGTGTCAGAGGTTTTCACCGTCATCACCCAAACAGCACAACGAATGAGG | Rev oligo for amplification of CvFAP/hphNT1 cassette with overlapping homologous region to p15018 |
| o13161 | CAGACAGGAGGCTAACTTCGTATAGCATACATTATACGAACGGTAACCAGAGACGGGTTGGC | Fwd oligo for amplification of CvFAP cassette with overlapping homologous region to p33001 |
| o13162 | GTTCTCAATCGCATGATGCTTGATCTATTAATAAATACACGGCTGTTTGCCACCTACAAGCCAG | Rev oligo for amplification of CvFAP cassette with overlapping homologous region to p33001 |
| o13164 | GAAGTTATGCCTCCTGTCTGACTC | Fwd oligo for amplification of backbone from p33001 |
| o33262 | GCAGCCGTGTATTTATTAATAGATCAAG | Rev oligo for amplification of backbone from p33001 |
| o13055 | CTTTCTCCACACCCCACAAAAAGACCCGTGCAGGACATCCTACTGCGTTTGCCACCTACAAGCCAGATTTTC | For sequencing of corresponding *CvFAP* CDS in *ALK1* locus |
| o13121 | GGAGGTTAAGAGAATTATCACCGG | For sequencing of corresponding *CvFAP* CDS in *ALK1* locus |
| o13034 | CGCTCTCTATATACACAGTTAAATTACATATCCATAGTCTAACCTTTACACCCACTCGTGCAGG | For amplification of corresponding *URA3* CDS |
| o13136 | GCAAGGCTACTATCGGTGCT | For amplification of corresponding *CvFAP* cassette in *ALK1* locus |
| o13139 | CAACGCTACCCTGTACCACC | For sequencing of corresponding *CvFAP* CDS in *ALK1* locus |
| o13140 | TGAACATTGACCAGGCTGCT | For sequencing of corresponding *CvFAP* CDS in *ALK1* locus |
| o11434 | GTCCCCGAATTACCTTTCCTC | For sequencing of corresponding *CvFAP* CDS in *ALK1* locus |
| o11065 | GTGTTTCTCGATACGGAGATGC | For amplification of corresponding *URA3* integration cassette in *ALK1* locus |
| o07024 | GTAATACACTGGGGAGAGGAC | For amplification of corresponding *CvFAP/URA3* integration cassette in *ALK1* locus |
| o07025 | AAGGAATTGTCTGACTTGCAAC | For amplification of corresponding *CvFAP/URA3* integration cassette in *ALK1* locus |
| o13157 | ATGCGAGCCTCTGCTGTC | For amplification of corresponding *CvFAP* CDS |
| o13158 | CTAAGCAGCCACAGTAGCAGG | For amplification of corresponding *CvFAP* CDS |
| o11084 | GCCCCAGATAAGGTTCCG | For sequencing of corresponding cleavage site of (CRISPRyl)-Hyg-URA3 (p94001) to confirm partial deletion of *URA3* |
| o15105 | GATCACATTTTTGTCGGCAAAGG | For amplification of corresponding *URA3* CDS |
| 94007 | CTTGCTATTTCTAGCTCTAAAACCGAGCTTGAGCACTCGAGCGAGGTCAACCTGCGCCGAC | 94007_CRISPRyl-URA3_for |
| 94008 | CCGGGTCGGCGCAGGTTGACCTCGCTCGAGTGCTCAAGCTCGGTTTTAGAGCTAGAAATAGCAAGTTAAAATAA | 94008_CRISPRyl-URA3_rev |

**Table S6: List of constructed vectors.**

Oligonucleotides for vector construction are listed in Tab. S5.

| **Designation** | **Genotype** | **Description** | **Source** |
| --- | --- | --- | --- |
| p13001 | CEN1-2, ARS68 region, pUC1, kanR, ampR, *pTEF1, YlCvFAP, tXPR2*, hphNT2 | Replicative, CvFAP-overepxression vector | This work |
| p55001 | pCRISPRyl , Ars68-  CEN, AmpR, Codon optimized Cas9 from *S. pyogenes* | pCRISPRyl with hygromycin marker | This work, derived from CRISPRyl, Addgene #70007,see material & methods |
| p94001 | p55001, Insert sgRNA (in URA3):  CGCTCGAGTGCTCAAGCTC | pCRISPRyl-Hyg-URA3 | This work |
| p13012 | *pTEF1 CvFAP XPR2t*  *loxP66 URA3 loxP71* | Integrative CvFAP/URA3 expression vector with *ALK1* homology arms | This work |

**Fig. S8: Light emission of LED-device.**

Light emission of advertised distinct 465-470 nm LED-light strip by wavelengths. The defined spectrum was verified by broad range spectrometer LR1-T from ASEQ instruments.


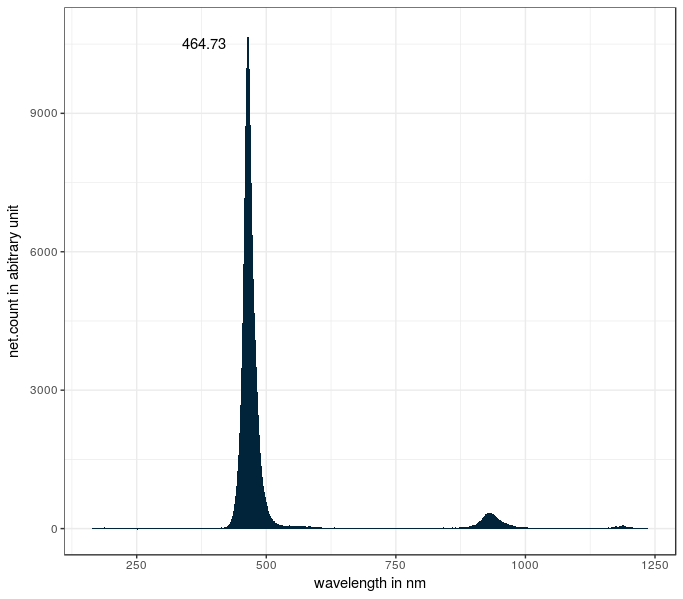


**Table S7: Correlation of light intensity and power supply values.**

Single LED measurements to determine technical conditions for bioprocess cultivation. The applied voltage was limited to a maximum of 12 V. The LED-strip was connected to the power-supply Consort EV231 (purchased from Sigma Aldrich). Photosynthetically active radiation (PAR) was measured using a Li-190SA quantum sensor (Li-COR) coupled with a Li-1000 (Datalogger).

| **Intensity**  **[µmol photons s^-1^ m^-2^]** | **Measuring Error**  **+/-** | **Amperage**  **[mA]** |
| --- | --- | --- |
| 68 | 8 | 100 |
| 130 | 9 | 200 |
| 192 | 9 | 300 |
| 250 | 9 | 400 |
| 310 | 9 | 500 |
| 357 | 10 | 600 |
| 415 | 10 | 700 |
| 455 | 11 | 800 |
| 533 | 11 | 900 |
| 545 | 12 | 1000 |

**Fig. S9 Hydrocarbon peaks vs background.**

Measurement procedure is fully described in material and methods section (5.7).

**A:** Assigned peaks of pentadecane (C15:0), 7-pentadecene (C15:1),


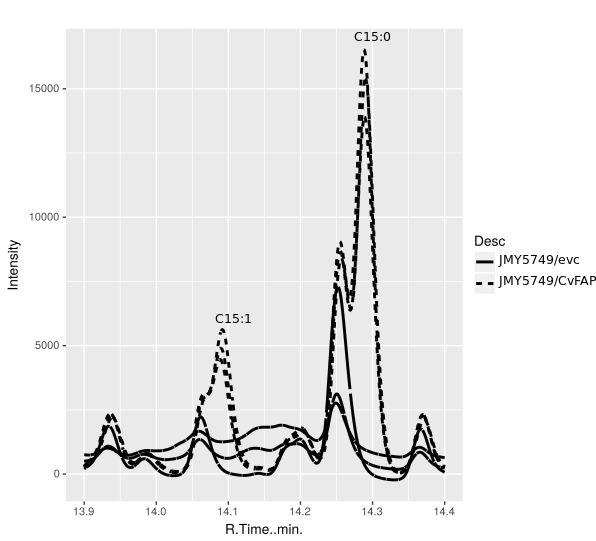


**B:** heptadecane (C17:0) 8-heptadecene (C17:1) and 6,9-heptadecadiene (C17:2) are shown for analysis of cell extraction of alkane producing strain JMY5749/CvFAP in comparison to the empty vector control.


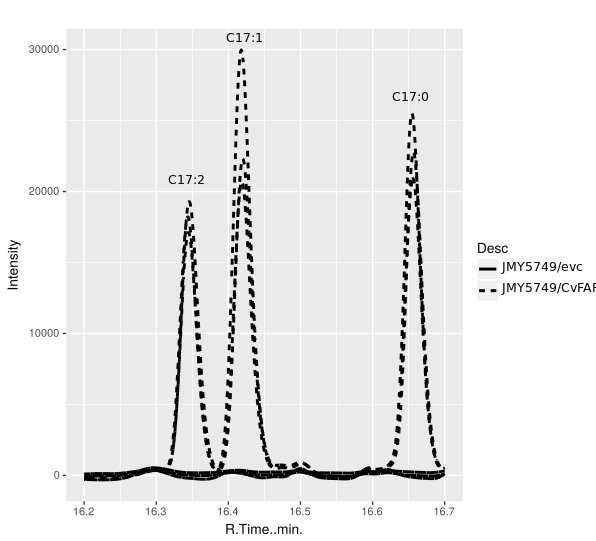


**References**

The PyMOL Molecular Graphics System, Version 2.0 Schrödinger, LLC.

[1. Kelley LA, Mezulis S, Yates CM, Wass MN, Sternberg MJE. The Phyre2 web portal for protein modeling, prediction and analysis. Nature Protocols. 2015. p. 845–58. Available from:](http://paperpile.com/b/yM8L7g/TspL) <http://dx.doi.org/10.1038/nprot.2015.053>

[2. Arnoux P, Sorigue D, Beisson F, Pignol D. Structure of Fatty acid Photodecarboxylase in complex with FAD and palmitic acid [Internet]. 2017. Available from:](http://paperpile.com/b/yM8L7g/d6vD) <http://dx.doi.org/10.2210/pdb5ncc/pdb>
